# Significant contrasts in aerosol acidity between China and the United States

**DOI:** 10.5194/acp-21-8341-2021

**Published:** 2021-06-01

**Authors:** Bingqing Zhang, Huizhong Shen, Pengfei Liu, Hongyu Guo, Yongtao Hu, Yilin Chen, Shaodong Xie, Ziyan Xi, T. Nash Skipper, Armistead G. Russell

**Affiliations:** 1 School of Civil and Environmental Engineering, Georgia Institute of Technology, Atlanta, Georgia 30332, USA; 2 School of Environmental Science and Engineering, Southern University of Science and Technology, Shenzhen, Guangdong, 518055, China; 3 School of Earth and Atmospheric Sciences, Georgia Institute of Technology, Atlanta, Georgia 30332, USA; 4 Department of Chemistry, University of Colorado, Boulder, Colorado 80309, USA; 5 Cooperative Institute for Research in Environmental Sciences, University of Colorado, Boulder, Colorado 80309, USA; 6 College of Environmental Sciences and Engineering, State Key Joint Laboratory of Environmental Simulation and Pollution Control, Peking University, Beijing, 100871, China

## Abstract

Aerosol acidity governs several key processes in aerosol physics and chemistry, thus affecting aerosol mass and composition and ultimately climate and human health. Previous studies have reported aerosol pH values separately in China and the United States (USA), implying different aerosol acidity between these two countries. However, there is debate about whether mass concentration or chemical composition is the more important driver of differences in aerosol acidity. A full picture of the pH difference and the underlying mechanisms responsible is hindered by the scarcity of simultaneous measurements of particle composition and gaseous species, especially in China. Here we conduct a comprehensive assessment of aerosol acidity in China and the USA using extended ground-level measurements and regional chemical transport model simulations. We show that aerosols in China are significantly less acidic than in the USA, with pH values 1–2 units higher. Based on a proposed multivariable Taylor series method and a series of sensitivity tests, we identify major factors leading to the pH difference. Compared to the USA, China has much higher aerosol mass concentrations (gas + particle, by a factor of 8.4 on average) and a higher fraction of total ammonia (gas + particle) in the aerosol composition. Our assessment shows that the differences in mass concentrations and chemical composition play equally important roles in driving the aerosol pH difference between China and the USA – increasing the aerosol mass concentrations (by a factor of 8.4) but keeping the relative component contributions the same in the USA as the level in China increases the aerosol pH by ~1.0 units and further shifting the chemical composition from US conditions to China’s that are richer in ammonia increases the aerosol pH by ~0.9 units. Therefore, China being both more polluted than the USA and richer in ammonia explains the aerosol pH difference. The difference in aerosol acidity highlighted in the present study implies potential differences in formation mechanisms, physicochemical properties, and toxicity of aerosol particles in these two countries.

## 1 Introduction

As an intrinsic aerosol property, aerosol acidity (usually characterized by aerosol pH) plays an important role in a variety of aerosol physical and chemical processes (Pye et al., 2020). Aerosol acidity can modulate aerosol mass by controlling the gas–particle partitioning of volatile and semi-volatile acids (such as $HCl-{Cl}^{-}$ and ${HNO}_{3}-{NO}_{3}^{-}$) (Guo et al., 2016) and can influence production rates of secondary aerosols through heterogeneous pathways (Jang et al., 2002; Surratt et al., 2010; Pathak et al., 2011). Acidity also affects aerosol optical properties via proton dissociation of organic functional groups (Mo et al., 2017) and the morphology or phase state of organic aerosols (Losey et al., 2016, 2018). Recent evidence links aerosol acidity to aerosol toxicity and health outcomes. For example, highly acidic aerosols cause greater dissolution of metals which can generate reactive oxygen species in vivo (Fang et al., 2017). High aerosol acidity is associated with increased risks of respiratory disease and cancer (Kleinman et al., 1989; Gwynn et al., 2000; Behera et al., 2015).

Due to the difficulties in directly measuring aerosol pH (Jang et al., 2002; Li and Jang, 2012), thermodynamic models, including ISORROPIA II (Fountoukis and Nenes, 2007), E-AIM (Clegg et al., 1998), and SCAPE2 (Kim and Seinfeld, 1995), have been widely used to calculate aerosol pH based on measured gaseous and particle composition and meteorological data such as relative humidity (RH) and temperature. Multiple studies suggest that these models can reproduce the partitioning of semi-volatile species including ${HNO}_{3}-{NO}_{3}^{-}$ and ${NH}_{4}^{+}-{NH}_{3}$, which are sensitive to aerosol pH (Guo et al., 2015, 2016; Hennigan et al., 2015).

Analyses of field observations in different regions of the United States (USA) have indicated that aerosol acidity is typically high. For example, Weber et al. (2016) showed that aerosol pH in the southeastern USA was buffered to be consistently in the range of 0–2 despite a substantial sulfate reduction over the past 15 years, and the same trend may be applicable to other regions. Studies in the northeastern USA and California also found highly acidic aerosols with mean pH values of 0.8 and 1.9, respectively (Guo et al., 2017a). Aerosol pH in the midwestern USA was typically higher than in other areas, with an average of 3.8 (Lawal et al., 2018). Studies in China, on the other hand, have found generally higher aerosol pH. Several studies in the heavily polluted North China Plain (NCP) region reported average pH of 3.5–5.2 (Shi et al., 2017, 2019; Ding et al., 2019; Song et al., 2019; Wang et al., 2020). Xi’an, a city in northwest China, had aerosol pH values of up to 5 (Wang et al., 2016; Guo et al., 2017b). Some sites in southeast China had lower aerosol pH, such as the site in Guangzhou which had an average of 2.3 (Jia et al., 2020). A comprehensive, nationwide comparison of aerosol pH between China and the USA can provide a better understanding of the factors driving aerosol pH and its effect on aerosol formation mechanisms and properties (Pathak et al., 2009; Guo et al., 2017a; Wang et al., 2020). However, such comparisons are still scarce (Guo et al., 2017b; Nenes et al., 2020; Zheng et al., 2020), primarily because of a lack of extensive simultaneous measurements of aerosol composition and semi-volatile gaseous compounds in China.

In this study, we compared the aerosol mass concentrations, chemical composition, and acidity between China and the USA based on 1-year measurements from 34 ground monitoring sites in the USA and 16 sites in China (mostly clustered in the NCP). In order to extend the spatial coverage to nationwide scales, we employed the Community Multiscale Air Quality (CMAQ) model to simulate the concentrations of gaseous and particle species which were used to calculate aerosol pH across both countries. We propose a new method to identify the factors driving the pH difference between these two countries and discuss the causes and implications of the pH difference.

## 2 Data collection and method

### 2.1 Observational data

Measurements of gaseous species (including ${HNO}_{3},\;{NH}_{3}$, and HCl) and particle components (including ${SO}_{4}^{2-},\;{NO}_{3}^{-},\;{NH}_{4}^{+},\;{Cl}^{-}$, and nonvolatile cations (NVCs)) from monitoring networks in China and the USA are used for analysis and comparison in this study. NVCs considered are ${Na}^{+},\;{Mg}^{2+},\;K^{+}$, and ${Ca}^{2+}$. The names and locations of the monitoring sites can be found in Tables S1 and S2. The sum of total observed aerosol ionic compounds is defined as water-soluble ions (WSIs), though it is recognized that not all of the ions are routinely measured (e.g., trace species and organic ions). We also study the partitioning of semi-volatile species including ${NH}_{3}-{NH}_{4}^{+}$ and ${HNO}_{3}-{NO}_{3}^{-}$ because they are sensitive to pH, especially when the partitioning ratios, $\varepsilon \left({NH}_{4}^{+}\right)$ and $\varepsilon ({NO}_{3}^{-})$, defined as the molar ratio of ${NH}_{4}^{+}$ to total ammonia $\left({TNH}_{3}={NH}_{3}+{NH}_{4}^{+}\right)$ and the molar ratio of ${NO}_{3}^{-}$ to total nitrate $\left({TNO}_{3}={HNO}_{3}+{NO}_{3}^{-}\right)$, are around 50% (Guo et al., 2017a; Chen et al., 2019).

In the USA, observational data are from co-located Clean Air Status and Trends Network (CASTNET) (https://www.epa.gov/castnet, last access: 23 January 2021) and Ammonia Monitoring Network (AMoN) (http://nadp.slh.wisc.edu/amon/, last access: 23 January 2021) sites. CASTNET and AMoN sites are assumed to be co-located if they are within 1 km of one another. Observations from co-located sites are then combined for pH calculation. Weekly ambient concentrations of gases and particulate species, including ${HNO}_{3},\;{SO}_{4}^{2-},\;{NO}_{3}^{-},\;{NH}_{4}^{+},\;{Cl}^{-}$, and NVCs, are available from CASTNET sites, while biweekly concentrations of ${NH}_{3}$ are available from AMoN sites. To match biweekly data of ${NH}_{3}$ from AMoN to weekly data of other species from CASTNET, the same ${NH}_{3}$ data are used for both weeks of the CASTNET samples. This assumption is expected to have a minor effect on pH estimates, as a previous study found that a 10-times increase in ${NH}_{3}$ is required to increase pH by 1 unit (Guo et al., 2017b). This assumption is also supported in later discussion (Sect. S1). HCl data are not available, so we use particle-phase ${Cl}^{-}$ as total Cl for pH calculations. Sensitivity tests assuming HCl concentrations of 4 times the ${Cl}^{-}$ concentrations or using HCl concentrations derived from CMAQ-modeled $HCl/{Cl}^{-}$ ratios show little difference in aerosol pH compared to the pH estimated by using particle-phase ${Cl}^{-}$ as total Cl (Fig. S1). Considering the small reported change in aerosol pH in the USA over a long-term period (Weber et al., 2016; Lawal et al., 2018) and the configuration of the chemical transport model which is set up for the year 2011 (see the following section), we use observational data in 2011 to investigate the aerosol pH in the USA. Only sites with measurements available for all species were selected for this study. There are 34 co-located CASTNET and AMoN sites, which are scattered across the contiguous USA (Figs. 3, S2a). The accuracy of CASTNET measurements has been assessed through the analysis of reference and continuing calibration verification samples with a criterion of 95%–105% (except ${NH}_{4}^{+}$, whose accuracy criterion is 90%–110%). Detailed information about data quality is available in the 2011 annual *CASTNET Quality Assurance Report* (United States Environmental Protection Agency, 2012a). A previous study demonstrated that the ${NH}_{3}$ concentrations measured by the passive AMoN samplers are comparable to annular denuder systems (as a reference system) with a mean relative percent difference of −9% (Puchalski et al., 2015).

In China, hourly observational data are extracted from the data-sharing platform operated by the Comprehensive Observation Network for Air Pollution in Beijing-Tianjin-Hebei and Its Surrounding Areas (http://123.127.175.60:8765/siteui/index, last access: 18 November 2019). This collaborative observation network is supported by multiple institutions and provides simultaneous observations of gaseous and particle species at individual monitoring sites (Wang et al., 2019). We derive daily average concentrations of gaseous species including ${NH}_{3},\;{HNO}_{3}$, and HCl and of particle species including ${NH}_{4}^{+},\;{NO}_{3}^{-},\;{Cl}^{-}$, and NVCs from hourly observational data at 16 monitoring sites for use in pH calculation. These monitoring sites are clustered in the NCP in eastern China (Fig. S2c). Due to the lack of data quality information, we first process the data by removing unreasonable data points. We define a set of valid data containing all the measured components in 1d as one case. We first remove cases with one or more missing components. In this step, 2704 of 5840 cases are removed. We then identify data points that are more than 3 median absolute deviations from the median as outliers and remove cases with any component identified as an outlier. Eventually, 1766 cases remain for subsequent analyses. Although we remove many cases in this process, the remaining cases cover most of the days in a year and are evenly distributed by month (Table S3).

It should be noted that the weekly (or longer) duration of the CASTNET samples in the USA may lead to biases in the measured concentrations especially for volatile species such as ammonium nitrate. Sickles et al. (1999) conducted a comprehensive comparison of measurements using the CASTNET weekly-duration sampling approach with those using a 24 h duration sampling approach. Both approaches used filter packs. They found that compared to 24 h sampling, weekly sampling led to low biases of −5 %, −5 %, and −0.7 %, on average, in measured ${HNO}_{3},\;{NO}_{3}^{-}$, and ${NH}_{4}^{+}$, respectively, and high biases of 4% and 16%, on average, in ${SO}_{4}^{2-}$ and ${SO}_{2}$, respectively. To evaluate the potential biases in the calculated aerosol pH due to the weekly-duration sampling, we conduct a sensitivity test to adjust the CASTNET-measured concentrations based on the reported average differences between weekly-duration and 24 h duration samples (Sickles et al., 1999) (Sect. 3).

### 2.2 Model configuration

We use CMAQ version 5.0.2 (United States Environmental Protection Agency, 2014) to simulate gaseous and particle species concentrations and aerosol pH in China and the USA. The model domains cover mainland China and the contiguous USA with 124×184 and 112×148 horizontal grid cells, respectively. Both are resolved at the 36 km horizontal resolution with 13 vertical layers extending to ~16 km above the ground. In both simulations, gas-phase chemistry is modeled with the CB05 chemical mechanism (Yarwood et al., 2005), and the aerosol thermodynamic equilibrium is modeled with ISORROPIA II (Fountoukis and Nenes, 2007).

The meteorological and emission inputs used to drive the China simulation are adopted from “AiMa”, an online operational air quality forecasting system (Lyu et al., 2017; AiMa Forecast, 2017). In the AiMa modeling system, the meteorological data are generated with the Weather Research and Forecasting (WRF) model (Skamarock et al., 2008) driven by the 0.5° global weather forecast products produced by the National Centers for Environmental Prediction (NCEP) Global Forecast System (GFS) model. The AiMa emission inventory was compiled and derived by integrating a variety of inventories and utilizing various activity data and has been continuously updated since its establishment (Lyu et al., 2017). The base year of the current AiMa emission inventory is 2017. For the US simulation, we use WRF-modeled meteorological fields downscaled from the North American Regional Reanalysis (NARR) data (Mesinger et al., 2006) as the meteorological input and the 2011 National Emissions Inventory provided by the US Environmental Protection Agency as the emission input (United States Environmental Protection Agency, 2012b). The base year of the meteorology and emissions is consistent with the year of the measurements in each country (i.e., 2017 for China and 2011 for the USA).

In order to evaluate the model performance against observations, we calculate normalized mean bias (NMB) and normalized root-mean-square error (NRMSE) to evaluate the spatial variation in pH, species concentrations, and partitioning ratios with the following equations:

(1) $$NMB=\frac{\sum_{1}^{N}\;\left(C_{m}-C_{o}\right)}{\sum_{1}^{N}C_{o}},$$

(2) $$NRMSE\;=\frac{\sqrt{\frac{\sum_{1}^{N}{\left(C_{m}-C_{o}\right)}^{2}}{N}}}{{\overset{¯}{C}}_{o}},$$

where $C_{m}$ is the modeled value, $C_{o}$ is the observed value, and N is the number of simulation–observation pairs.

### 2.3 Aerosol pH calculation

In this study, we use the ISORROPIA II thermodynamic model (Fountoukis and Nenes, 2007) to determine the composition in a $K^{+}-{Ca}^{2+}-{Mg}^{2+}-{NH}_{4}^{+}-{Na}^{+}-{SO}_{4}^{2-}-{NO}_{3}^{-}-{Cl}^{-}-H_{2}O$ aerosol system under equilibrium conditions with gas-phase precursors. Aerosol pH is calculated based on $H_{air}^{+}$ and liquid water content (LWC) from ISORROPIA II output:

(3) $$pH=-{log}_{10}\left(\gamma_{H^{+}}\cdot H_{aq}^{+}\right)=-{log}_{10}\left(\frac{1000\gamma_{H^{+}}\cdot H_{air}^{+}}{LWC}\right),$$

where $\gamma_{H^{+}}$ is the activity coefficient of the hydronium ion which is assumed to be 1 in this study (the binary activity coefficients of ionic pairs, including $H^{+}$, are calculated in ISORROPIA II), $H_{aq}^{+}\;(mol\;L^{-1})$ is the hydronium ion concentration in aerosol liquid water, and $H_{\text{air}\;}^{+}\;\left(\mu g\;m^{-3}\right)$ is the equilibrium particle hydronium ion concentration per volume air. LWC $\left(\mu g{\;m}^{-3}\right)$ in this study only considers the water uptake by inorganic species. The effect of water uptake by organic species on aerosol pH has been found to be minor (Guo et al., 2015).

There are two modes in ISORROPIA II’s calculation: the forward mode and the reverse mode. In the forward mode, the inputs include total concentrations (i.e., gas + particle) of ${TNH}_{3},\;{TNO}_{3},\;TCl\;\left(HCl+{Cl}^{-}\right),\;{SO}_{4}$, NVCs, and meteorological parameters (temperature and RH); in the reverse mode, only the particle phase of compounds and meteorological parameters are needed (Fountoukis and Nenes, 2007). In this study, the ISORROPIA II model is run in the forward mode for aerosols in a metastable state because the concentrations of both gas and particle species are available and also because the reverse mode has been reported to be more sensitive to measurement errors (Hennigan et al., 2015; Song et al., 2018).

We find that there are measurements with unrealistically high ${Ca}^{2+}$ concentrations (such that ${Ca}^{2+}$ is more than LWC × 0.002, i.e., the solubility of ${Ca}^{2+}$ in aerosol liquid water). This may be due to the measurement method of ${Ca}^{2+}$ which uses large amounts of water to dissolve filter-collected particles. This process will likely dissolve the water-insoluble part of ${Ca}^{2+}$ in aerosols which may cause higher bias in aerosol ${Ca}^{2+}$ concentrations. In the existence of aerosol ${SO}_{4}^{2-},\;{Ca}^{2+}$ precipitates along with ${SO}_{4}^{2-}$ as ${CaSO}_{4}$ because of the low solubility (Seinfeld and Pandis, 2006). Including the high ${Ca}^{2+}$ concentration leads to large differences in estimated pH because of the high acidity of ${SO}_{4}^{2-}$ (Sect. S2). In order to avoid this potential bias, we use a modified ${Ca}^{2+}$ concentration for pH calculations while keeping ${SO}_{4}^{2-}$ unchanged. That is, we use the original ${Ca}^{2+}$ concentration to calculate aerosol LWC and then use the concentration of ${Ca}^{2+}$ that can dissolve in the LWC as the modified ${Ca}^{2+}$ concentration in cases where the original ${Ca}^{2+}$ exceeds its solubility in the calculated LWC.

We compare the directly measured gas–particle partitioning ratios of semi-volatile compounds with the ratios repartitioned by ISORROPIA II using measured total (gas + particle) concentrations as inputs. The purpose of this comparison, as conducted in previous studies (Guo et al., 2016, 2017a), is to examine the measurement data quality. This method is effective when the species have substantial fractions in both gas and particle phases (Guo et al., 2017a). The comparison results of $\varepsilon ({NH}_{4}^{+})$ and $\varepsilon ({NO}_{3}^{-})$ are shown in Fig. S3. The correlation coefficients and the slopes of linear regression are all close to 1, suggesting good agreement between the measured and ISORROPIA-re-calculated partitioning ratios. In terms of these partitioning ratios, the model (ISORROPIA II) performs better in the USA than in China, which may be attributable, in part, to the more balanced partitioning of the species between gas and particle phases in the USA.

### 2.4 Multivariable Taylor series method (MTSM)

To separate the contribution of individual components (eight species in total, including ${Na}^{+},\;{SO}_{4},\;{TNO}_{3},\;{TNH}_{3},\;TCl,\;{Ca}^{2+},\;K^{+}$, and ${Mg}^{2+}$) and meteorological variables (RH and temperature) to the pH difference between China and the USA, we propose a multivariable Taylor series method (MTSM). First, we derive the average conditions (i.e., species concentrations and meteorological conditions) across all the sites in the USA and China. We then use the USA as the starting point and China as the end point and decompose the contributions of individual compounds to the pH difference based on the following equations:

(4) $$\Delta c_{i}=c_{i,\text{China}}-c_{i,US},$$

(5) $$c_{i,\lambda }≅c_{i,US}+\Delta c_{i}\cdot \lambda ,$$

(6) $$\Delta \text{pH}=pH_{\text{China}}-pH_{\text{US}}=\int_{0}^{1}\left(\sum_{i=1}^{10}\frac{\partial \text{pH}}{\partial c_{i,\lambda }}\cdot \Delta c_{i}\right)\cdot \text{d}\lambda \;\;\;\;\;\;\;\;\;\;\;≅\sum_{s=1}^{100}\left(\sum_{i=1}^{10}\frac{\partial \text{pH}}{\partial c_{i,\frac{s}{100}}}\cdot \Delta c_{i}\right)\cdot 0.01,$$

(7) $$\Delta {pH}_{i}≅\sum_{s=1}^{100}\frac{\partial pH}{\partial c_{i,\frac{s}{100}}}\cdot \Delta c_{i}\cdot 0.01,$$

where subscript i denotes a specific species or meteorological variable; $c_{i,\text{China}\;}$ and $c_{i,US}$ represent the values of i in China and the USA, respectively; $\Delta c_{i}$ is the difference in $c_{i}$ between China and the USA; $c_{i,\lambda }$ is an intervening $c_{i}$ between $c_{i,\text{China}\;}$ and $c_{i,\text{US}\;}$ defined by λ,λ∈[0,1]; when λ is 0, $c_{i,\lambda }$ is $c_{i,US}$; when λ is 1, $c_{i,\lambda }$ is $c_{i,\text{China}}$. The pH difference between China and the USA (i.e., ΔpH) can be expressed as the sum of the partial derivatives of pH with respect to $c_{i,\lambda }$ which is then integrated from $c_{i,US}$ to $c_{i,\text{China}}$, as described by Eq. (6). In this study, we take 100 steps with equal intervals to gradually change λ from 0 to 1 (Eq. 6) and record the partial derivatives of pH with respect to individual $c_{i,\lambda }$ and derive the contributions of all the species and meteorological variables to the pH change at every step. By summing up the contributions of individual variables at all steps, we characterize the contributions of individual factors to the overall pH difference (Eq. 7). Based on the same method, we further quantify the contributions of individual factors to the differences in LWC and $H_{\text{air}}^{+}$, respectively, the two variables directly used to calculate aerosol pH (Sect. 3).

## 3 Results and discussion

### 3.1 The pH difference between China and the USA

#### 3.1.1 The pH difference based on observations

**Figure 1.**
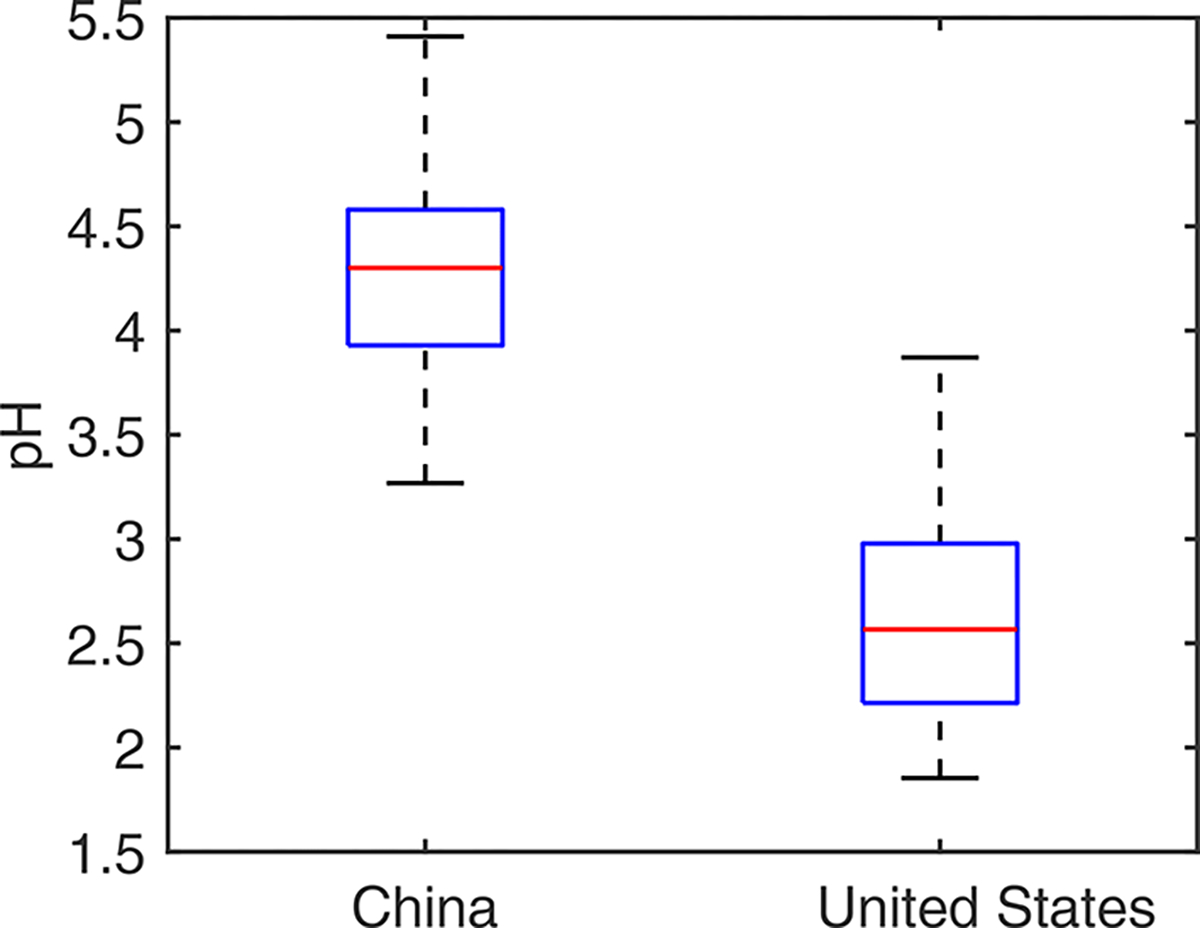
Annual average aerosol pH at each monitoring site in China and the United States based on observational data. The arithmetic mean (midline), the interquartile range (box), and the minimum–maximum range (whiskers) are shown in the box plot.

The sensitivity test to adjust the CASTNET-measured concentrations based on the reported average differences between weekly-duration and 24 h duration samples shows little difference between the unadjusted and adjusted pH values in the USA (2.69 ± 0.85 and 2.74 ± 0.83 on average for the unadjusted and adjusted pH, respectively), suggesting that the weekly duration of the CASTNET sampling has little impact on the calculated aerosol pH. Therefore, we proceed with our subsequent analyses using the unadjusted pH. The aerosol pH values calculated based on observational data show a significant difference between China (most observation sites are in the NCP) and the USA. In China (mainly the NCP), the 2017 annual average pH at monitoring sites is 4.3, ranging from 3.3 to 5.4 with an interquartile range of 3.9–4.6. In the contiguous USA, the 2011 annual average pH is 2.6, ranging from 1.9 to 3.9 with an interquartile range of 2.2–3.0 (Fig. 1). The t test shows a statistically significant difference between the two groups (p<0.0001), suggesting that the aerosols are on average more acidic at the monitoring sites in the contiguous USA than in China (NCP).

**Figure 2.**
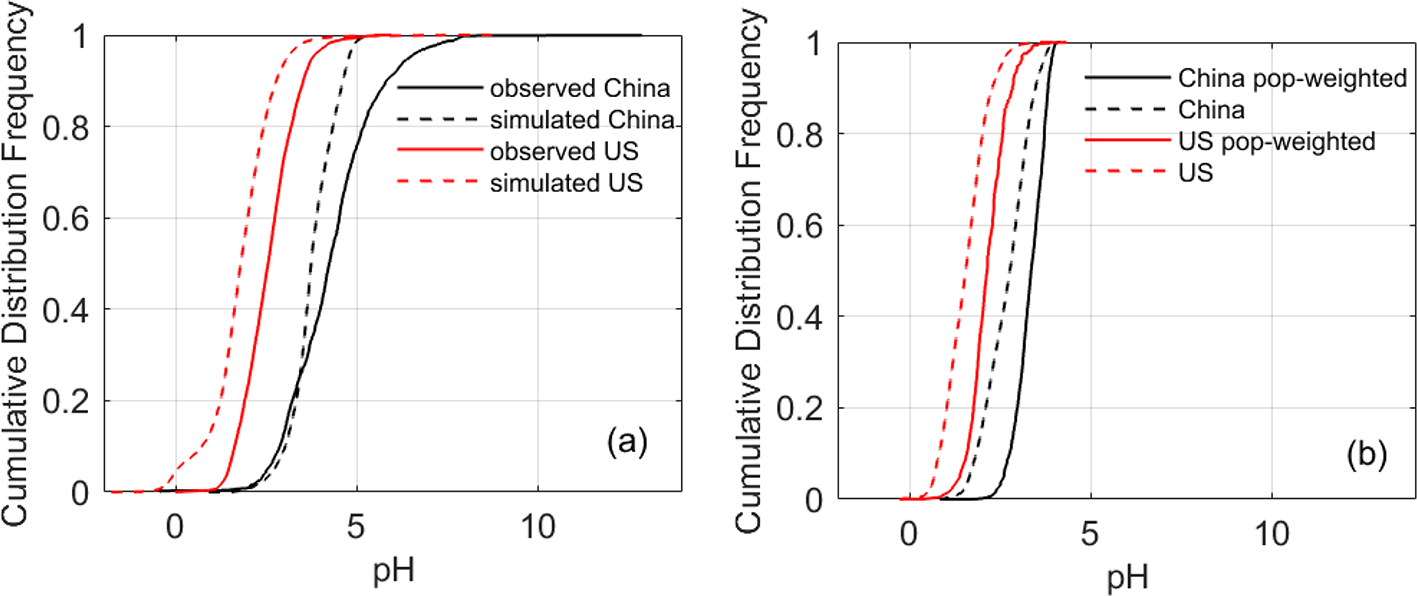
The cumulative distribution function (CDF) curves of aerosol pH in China and the United States based on (**a**) observed particulate and gaseous composition (solid lines) and CMAQ simulations collocated with observation sites (dashed line) and (**b**) simulated data nationwide. In panel (**b**), both average and population-weighted CDFs are shown.

The pH difference is also illustrated by the cumulative distribution function (CDF) curves (Fig. 2, solid lines). The shapes of the CDF curves are similar in these two countries with a slightly steeper slope in the contiguous USA (Fig. 2a). The pH values, however, are 1–2 units higher in China (NCP) than in the contiguous USA across varying levels of cumulative frequencies in the CDF curves. In some cases, aerosols could be completely neutral in China (NCP) (the frequency is 2% for pH≥7), while in the contiguous USA, the pH values in all cases were below 6.

Spatially, 14 out of the 16 sampling sites in China are in the NCP (Fig. S2c) which is one of the most populous and polluted regions in China (Hu et al., 2014; Cui et al., 2020). Our pH results in this region are consistent with those of other studies (ranging from 3.5 to 4.6) (Liu et al., 2017; Ding et al., 2019; Ge et al., 2019). The distribution of sampling sites in the USA, on the other hand, is more evenly distributed spatially. The pH values in the midwest and California are higher than in other regions like the southeast, in line with previous studies (Lawal et al., 2018; Chen et al., 2019). Overall, the pH level in the USA is 1.7 units lower than over the NCP of China.

#### 3.1.2 The pH difference based on model simulations

**Figure 3.**
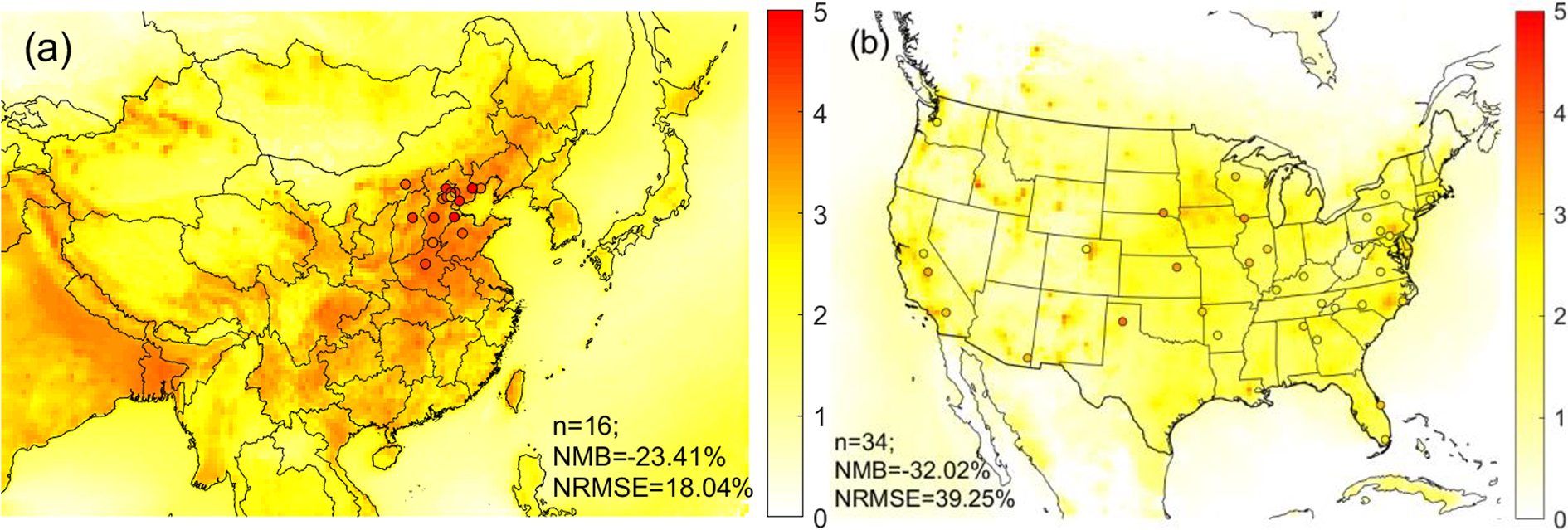
Overlay of annual mean pH calculated based on simulated concentrations (colored map) and observed concentrations (colored dots) over the study domain in (**a**) China and (**b**) the United States. Number of sites (N), normalized mean bias (NMB), and normalized root-mean-square error (NRMSE) are provided in each figure. The world shapefiles were obtained from Esri (ArcGIS Hub, Countries WGS84, http://www.arcgis.com/home/item.html?id=30e5fe3149c34df1ba922e6f5bbf808f, last access: 21 June 2019).

To address the issue of insufficient spatial coverage of the observational data in China, we conduct simulations using CMAQ, in conjunction with the observational data, to further study the pH difference on a nationwide scale. We evaluate the model performance by comparing the modeled and observed aerosol pH values (Fig. 3); major particle and gaseous species including ${SO}_{4}^{2-},\;{NO}_{3}^{-},\;{NH}_{4}^{+}$ and ${HNO}_{3}$, and ${NH}_{3}$; and the partitioning ratios including $\varepsilon \left({NH}_{4}^{+}\right)$ and $\varepsilon \left({NO}_{3}^{-}\right)$, at monitoring sites (Figs. S4–S6).

Spatially, the model simulations generally capture the observed variations in pH, species concentrations, and partitioning ratios, although there are some notable biases (Figs. S4 and S5). In both China (NCP) and the contiguous USA, the modeled ${NH}_{4}^{+},\;{NO}_{3}^{-}$, and ${NH}_{3}$ are biased low while modeled ${HNO}_{3}$ is biased high, resulting in low biases in the predicted $\varepsilon \left({NO}_{3}^{-}\right)$ and $\varepsilon \left({NH}_{4}^{+}\right)$. The modeled ${SO}_{4}^{2-}$ in both countries is biased low. Such low biases have been seen in previous studies (Fountoukis et al., 2013; Theobald et al., 2016) and have been attributed to the spatial mismatch between the observations and simulations due to the coarse resolutions of model grid cells (usually 20–50 km resolution) (Shen et al., 2014; R. Wang et al., 2014). Smaller NMBs in the USA indicate a better performance compared to China (NCP). Larger differences between observations and simulations in China (NCP) could also be caused by larger measurement uncertainties as the data in China are collected from different monitoring stations operated by individual research institutions (Wang et al., 2019) and thus lack unified quality control compared with data in the USA, which come from national monitoring networks (United States Environmental Protection Agency, National Atmospheric Deposition Program). The co-occurrence of low biases in $\varepsilon \left({NO}_{3}^{-}\right)$, which causes lower bias in aerosol pH, and low biases in $\varepsilon \left({NH}_{4}^{+}\right)$ and ${SO}_{4}^{2-}$, which cause higher bias in aerosol pH, likely offset each other, resulting in small biases in aerosol pH. Indeed, the simulated average pH values at observation sites (3.8 ± 0.2 in the NCP, China, and 1.8 ± 0.5 in the contiguous USA) are generally in line with the observed averages (4.3 ± 0.5 in the NCP, China, and 2.6 ± 0.5 in the contiguous USA) (Fig. 3), although the model shows a moderate low bias in both countries. The larger pH difference in the USA than in China is likely due to the low bias in ${TNH}_{3}$ to which the sensitivity of pH is found to be more pronounced in the USA than in China (discussed in detail in Sect. S1).

**Figure 4.**
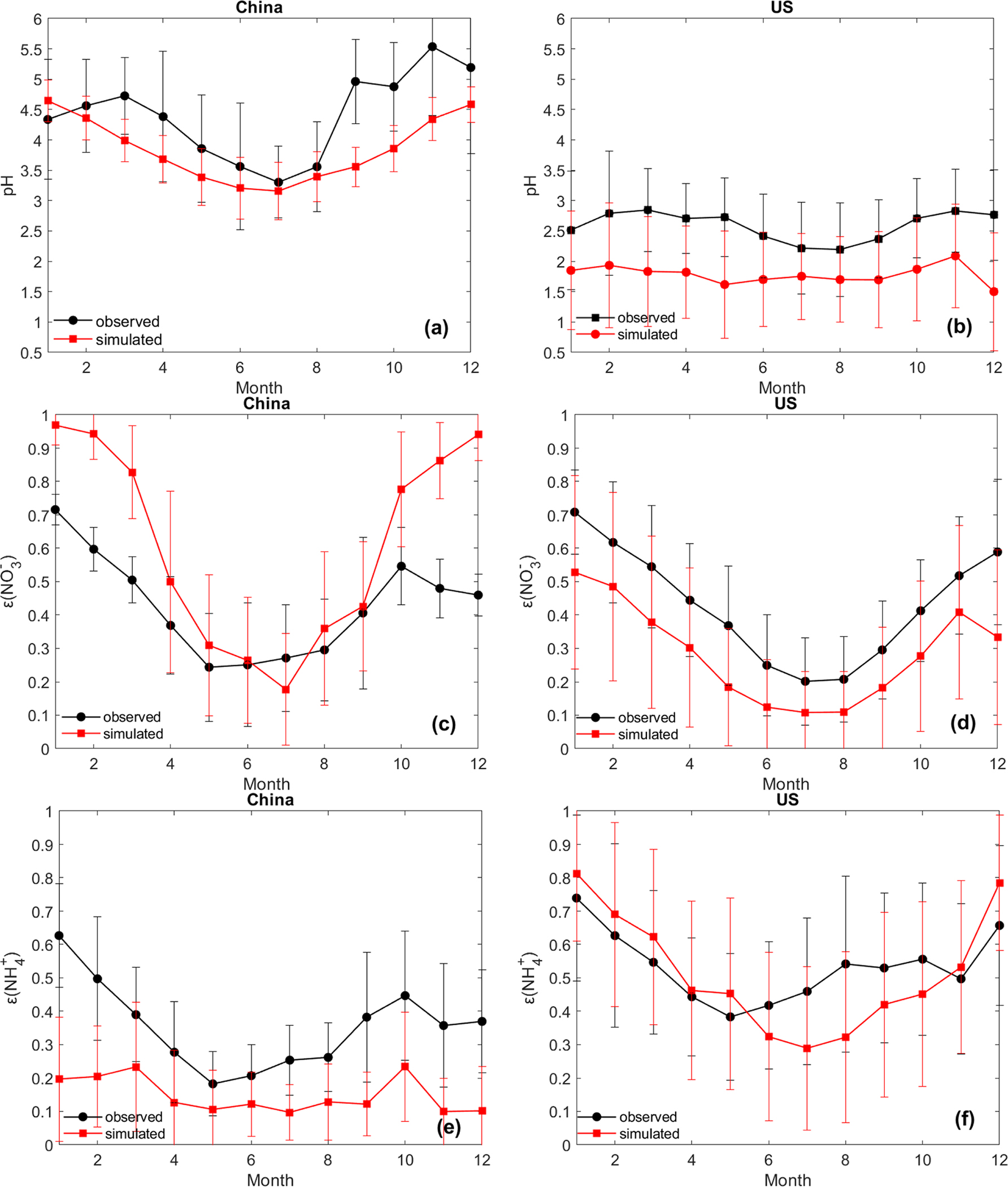
Monthly average values of pH, $\varepsilon \left({NO}_{3}^{-}\right)$, and $\varepsilon \left({NH}_{4}^{+}\right)$ based on observed and CMAQ-simulated data in China **(a, c, e)** and in the United States **(b, d, f)**. The error bars represent the standard deviation of all the cases in each month.

With respect to the temporal variation, the model captures the seasonal trends of pH, $\varepsilon \left({NH}_{4}^{+}\right)$, and $\varepsilon \left({NO}_{3}^{-}\right)$ in both countries, with lower values in summer and higher values in winter (Fig. 4). The lower temperature in winter favors the partitioning toward the particle phase for semi-volatile species. Comparison of the seasonal trends of the individual aerosol components shows a better agreement in the USA than in China. For example, the simulation in the USA captures the trends of almost all components, though it is biased low for ${SO}_{4}^{2-}$ and ${NH}_{4}^{+}$ in summer (Fig. S6b and h); the simulation in China misses the peaks of ${SO}_{4}^{2-}$ in winter and ${NH}_{3}$ in summer and has high biases for ${HNO}_{3}$ in summer (Fig. S6a, i, and e). Measurement-related biases may contribute to the disparity in the temporal trends between observed and modeled concentrations. The uncertainty in monthly profiles of emission estimates may also play an important role. For example, CASTNET’s long sampling period could lead to a larger measurement bias in summer than in winter (Sickles and Shadwick, 2008); the large uncertainty in the current estimates of ${NH}_{3}$ emissions in China, especially the reported underestimation of summertime emissions as indicated by an inversion analysis (Kong et al., 2019), may cause the absence of the summertime ${NH}_{3}$ peak in the simulated trend (Fig. S6i). Further investigation is needed to better understand the factors underpinning the disparity between observations and model simulations. In spite of the various potential uncertainties, overall, the spatial and temporal evaluation suggests generally good agreement between the model simulations and observations in both countries.

In line with observations (Sect. 3.1.1), the nationwide model simulations show significant differences in aerosol acidity between the two countries. Almost all the areas in the USA have aerosol pH values lower than 3 according to the CDF (Fig. 2b). Higher pH values are found in the middle and eastern USA, while in the western USA except California, the pH values are lower (Fig. 3). In China, a large portion of areas (87 %) have aerosol pH values above 3 according to the CDF. This is especially true in eastern China which has the largest population (Fig. 3). Aerosol pH values in western and southeastern China are generally lower than in the east. It should be noted that due to the scarcity of observational data, the pH estimates in southern and western China are not evaluated. The nationwide annual average pH values in China and the USA are 2.7 ± 0.6 and 0.8 ± 0.8 units, respectively, lower than the observation-based values due partly to the model bias but also because most of the monitoring sites are in areas with high pH (Fig. 3).

Given the adverse health impacts of ambient aerosols (Burnett et al., 2014; Freedman et al., 2019) and the potential linkage of aerosol acidity with aerosol toxicity through the solubility of redox-active metals (Oakes et al., 2012; Fang et al., 2015; Ye et al., 2018), we further calculate and compare the population-weighted averages of aerosol pH in the two countries to highlight the pH levels in densely populated areas. The population-weighted pH values are 3.3 ± 0.4 and 2.2 ± 0.5 in China and the USA, respectively, both of which are higher than non-weighted averages, indicating that aerosols in more populous areas tend to be less acidic (Fig. 2b). This finding is further confirmed by the statistically significant positive correlation (α=0.01), within each country, between the aerosol pH and population density (for China, r=0.42 and p<0.0001; for the USA, r=0.28 and p<0.0001). Consistent with the observation-based results, the t test for the model simulations shows a significant difference in both the population-weighted and non-weighted aerosol pH values between the two countries (p<0.001).

### 3.2 Causes of the aerosol pH difference

#### 3.2.1 Gaseous and particle compound profiles between China (NCP) and the contiguous USA

**Figure 5.**
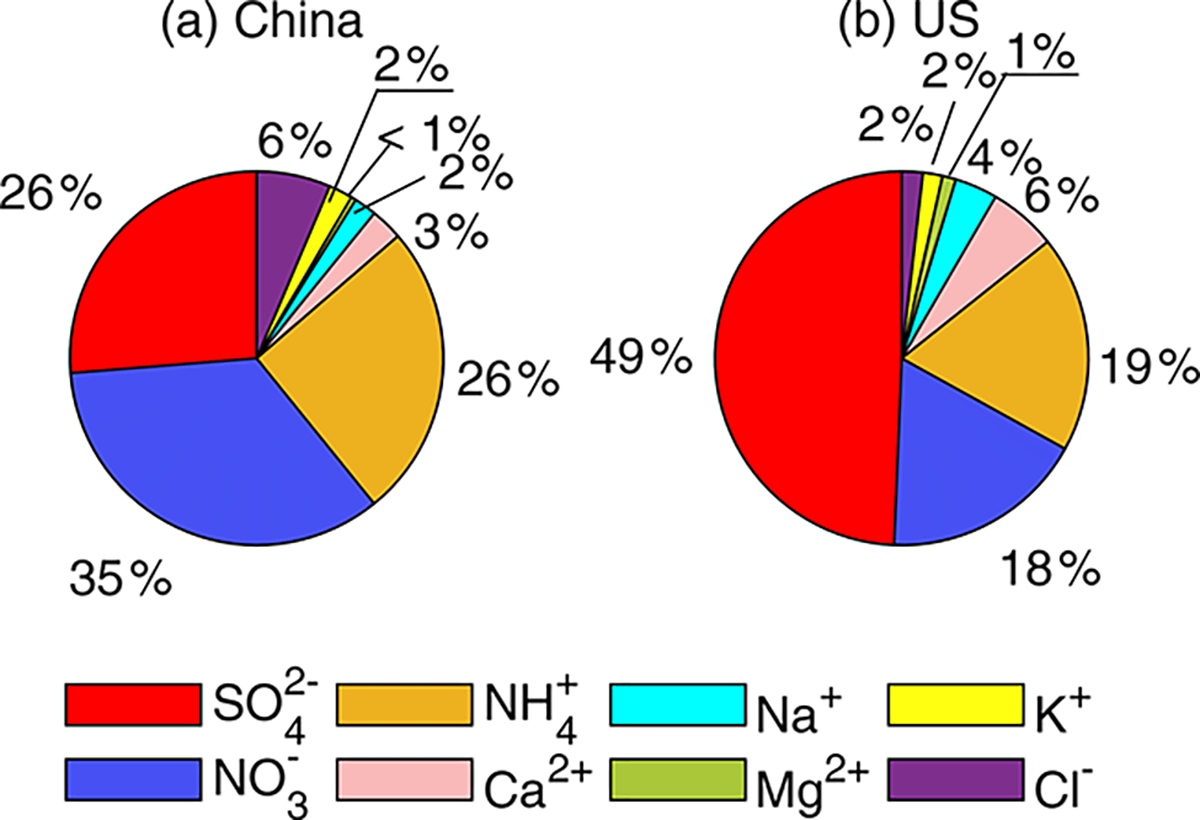
Annual average values of water-soluble ions (WSIs) concentration profiles in China (**a**) and in the United States (**b**).

We further investigate the factors leading to the pH difference. Although both observations and simulations are subject to uncertainty, we expect observations to provide more direct and reliable evidence for this investigation. It should be noted that the monitoring sites in China were clustered in the NCP and, thus, may not be representative of the whole of China. Table 1 summarizes the annual average concentrations of gaseous and particle species measured in China (NCP) and the contiguous USA during the study period (China, 2017; USA, 2011). For all the gaseous and ionic species (except ${HNO}_{3}$), the average concentrations in China (NCP) are statistically significantly higher than those in the contiguous USA. The total concentrations of WSI species in China (NCP) ($34.4\;\mu g\;m^{-3}$) are on average 6 times the concentrations in the contiguous USA ($5.7\;\mu g\;m^{-3}$) and have greater variation, ranging from $0.2-240\;\mu g\;m^{-3}$, compared to a range of $0.1-31\;\mu g\;m^{-3}$ in the contiguous USA. Similarly to in other studies in China (Yao et al., 2002; Pathak et al., 2009; Zhang et al., 2013; Liu et al., 2016) and the USA (Guo et al., 2015; Feng et al., 2020), ${NH}_{4}^{+},\;{NO}_{3}^{-}$, and ${SO}_{4}^{2-}$, contribute more than 80 % of the total WSI concentrations in both countries. The mass fractions of individual WSIs, however, differ between the two countries (Fig. 5). In China (NCP), the dominant WSI was ${NO}_{3}^{-}(34.6\%)$, followed by ${SO}_{4}^{2-}(26.3\%)$ and ${NH}_{4}^{+}(25.5\%)$. In the contiguous USA in 2011, ${SO}_{4}^{2-}$ contributed nearly half of the total WSI concentration (49.4%), and the contributions of ${NO}_{3}^{-}$ and ${NH}_{4}^{+}$ are comparable $\left({NO}_{3}^{-}17.6\%,{NH}_{4}^{+}18.8\%\right)$. Note that ${SO}_{4}^{2-}$ and ${NO}_{3}^{-}$ levels have been decreasing dramatically over the years, leading to decreases in ${NH}_{4}^{+}$ since there is less substrate to interact with ${NH}_{3}$ and form particulate ammonium species (Butler et al., 2016).

Two of the most predominant anions in aerosols, ${SO}_{4}^{2-}$ and ${NO}_{3}^{-}$, at the monitoring sites in China (NCP) are present at 4 and 15 times the concentrations, respectively, of those observed in the contiguous USA. The relative difference in ${NO}_{3}^{-}$ between the two countries is the most significant, compared with the differences in other WSI components. Hence, the difference in the nitrate-to-sulfate molar ratio $\left({NO}_{3}^{-}/{SO}_{4}^{2-}\right)$ is also significant between the two countries. The observational data show that the ratios at most monitoring sites in China (NCP) are larger than 1 and that only two sites have ratios lower than but close to 1 (0.81, 0.94). On the other hand, 27 out of the 34 sites in the contiguous USA show a ratio lower than 1, ranging from 0.25–0.99. High ${NO}_{3}^{-}/{SO}_{4}^{2-}$ in China (NCP) could be caused by more efficient oxidation of ${NO}_{x}$ than ${SO}_{2}$ in China leading to greater nitrate formation as well as higher aerosol pH and availability of ${NH}_{3}$ which favor the formation of particle nitrate (Guo et al., 2018b; Vasilakos et al., 2018). The varying ratios of ${NO}_{3}^{-}/{SO}_{4}^{2-}$ in aerosols could further affect aerosol liquid water uptake, which is discussed in the Supplement (Sect. S2).

The most abundant cation in aerosols is ${NH}_{4}^{+}$, and the concentration difference in ${NH}_{4}^{+}$ between the two countries (by a factor of 11) is more significant than the difference in other cations (by factors of 2–7). In addition, $\varepsilon \left({NH}_{4}^{+}\right)$ in China (NCP) (0.13–0.48) is approximately 50 % lower than in the contiguous USA (0.22–0.85), meaning that compared to the USA, ${TNH}_{3}$ in China tends to be present more in the gas phase. Higher ${NH}_{4}^{+}$ and lower $\varepsilon \left({NH}_{4}^{+}\right)$ levels in China amount to a higher level of ${TNH}_{3}$ which has an important influence on aerosol pH, partitioning of ${TNO}_{3}$, and even particulate mass (see Supplement for more discussion).

NVCs such as ${Na}^{+}$, ${Ca}^{2+}$, ${Mg}^{2+}$, and $K^{+}$ are often minor components of particles but are important because of their ability to neutralize acidic species in the atmosphere, such as sulfuric and nitric acids (Zhang et al., 2007). Neglecting NVCs would cause low biases in pH, driving the ${NH}_{3}-{NH}_{4}^{+}$ equilibrium to shift toward the particle phase because more ammonium would be used to neutralize the aerosols that would otherwise be neutralized by NVCs (Guo et al., 2018a). Therefore, NVCs are included in calculating aerosol pH in this study. High NVC concentrations usually occur at the sites near emission sources. For example, high concentrations of ${Na}^{+}$, mainly from sea salt (Zhang et al., 2011), occur at sites 13, 27, and 30 in the USA, which are all coastal sites. High concentrations of ${Ca}^{2+}$, mainly from mineral dust, are found at sites 6,11, and 23 in the contiguous USA and at Site 5 in China (NCP), which are in prairies impacted by sand and dust. Average NVC concentrations in China (NCP) are up to an order of magnitude higher than in the contiguous USA, although in both countries, most of the NVCs concentrations are small compared to those of ${SO}_{4}^{2-},\;{NO}_{3}^{-}$, and ${NH}_{4}^{+}$. The predominant NVCs in China (NCP) are ${Ca}^{2+}(2.8\%)$, $K^{+}(2.1\%)$, and ${Na}^{+}(2.0\%)$, and in the contiguous USA, they are ${Ca}^{2+}(5.9\%)$ and ${Na}^{+}(3.7\%)$.

#### 3.2.2 Characterization of contributions to aerosol acidity by individual factors

**Figure 6.**
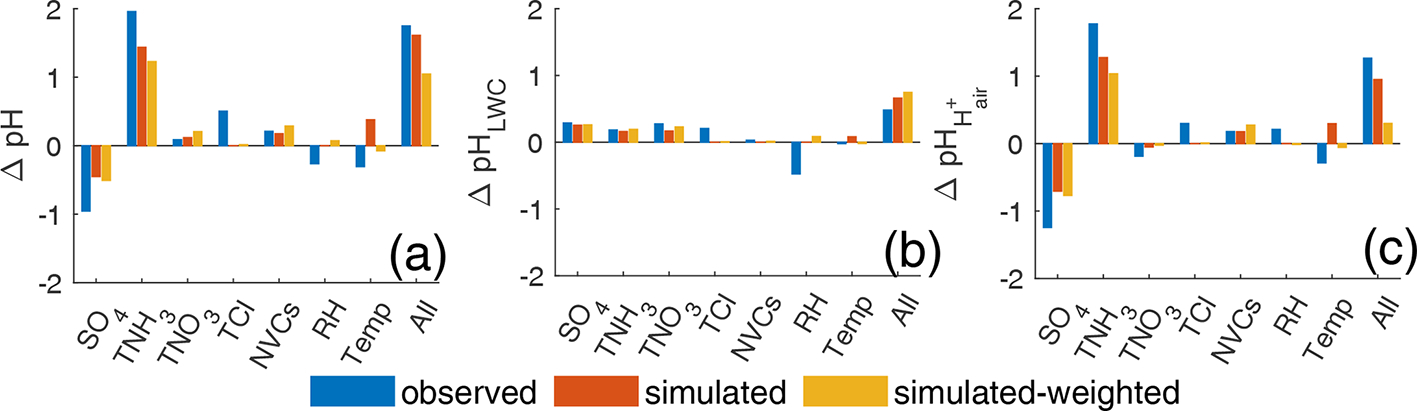
Contributions of individual components and meteorological factors to (**a**) total difference in aerosol pH(ΔpH), (**b**) the aerosol pH difference through the pathway of $LWC\;\left(\Delta {pH}_{LWC}\right)$, and (**c**) the aerosol pH difference through the pathway of $H_{air}^{+}(\Delta {pH}_{H_{air}^{+}})$ between China and the United States calculated by the multivariable Taylor series method (MTSM) described in Sect. 2.4. For each factor, the sum of the contributions through the two pathways yields the net contribution of this factor to the aerosol pH. The case in the United States is chosen as the starting point, and China is chosen as the ending point.

We use the MTSM as described in Sect. 2.4 to characterize the contribution of each component to the pH difference between the USA and China. Three groups (i.e., observation, simulation non-weighted, simulation population-weighted) of the annual average concentrations in the USA and China listed in Table S4 are chosen as the starting (USA) and ending (China) points to perform the analysis. The results are shown in Fig. 6.

The average concentrations based on the observational and simulated data are not completely consistent due to the representativeness of the monitoring sites and the discrepancy between the simulations and observations. The MTSM analyses based on the three groups, however, show similar results. For example, all three groups suggest the high ${TNH}_{3}$ in China as an important factor leading to the difference in aerosol pH between the two countries (Fig. 6). The contribution of ${TNH}_{3}$ is the highest in the observation group due to the large difference in ${TNH}_{3}$ concentration. Other cations, mainly NVCs, have a relatively small effect (0.2, 0.2, and 0.3 in groups “observation”, “simulation”, and “simulation-weighted”, respectively), which is consistent with a previous study (Zheng et al., 2020). Unlike ${TNH}_{3}$ and NVCs which lead to higher pH values in China than in the USA, ${SO}_{4}^{2-}$ contributes in the opposite direction to the pH difference. High ${SO}_{4}^{2-}$ concentrations decrease aerosol pH in China by 0.6–1.3 units, compared to the USA, although this effect is fully offset by ${TNH}_{3}$.

Compared to other species, the concentrations of ${TNO}_{3}$ are the most different between the two countries (by a factor of 15), but the MTSM shows that the contribution of ${TNO}_{3}$ to the pH difference is relatively small (0.1, 0.1, and 0.2 in the observation, non-weighted, and population-weighted groups). This result is further confirmed by a sensitivity test of ${TNO}_{3}$ (Fig. S10) which shows that the change in pH from changing only ${TNO}_{3}$ is small in both countries. More detailed analyses and discussions on the effects of ${TNH}_{3},\;{TNO}_{3}$, and ${SO}_{4}$ on aerosol pH can be found in the Supplement.

Studies have identified an important role of temperature in driving aerosol pH (Battaglia et al., 2017; Tao and Murphy, 2019; Jia et al., 2020). Our MTSM analysis shows that temperature accounts for 0.07–0.39 units of pH difference between China and the USA, which varies by group (Fig. 6). Such relatively small contributions of temperature, compared to those of ${TNH}_{3}$ and ${SO}_{4}$, are mainly because of the small difference in temperature between these two countries which are at similar latitudes. The difference in the annual average temperature between China and the USA is 1.4, −5.0, and 2.6 K in the observation, non-weighted, and population-weighted groups, respectively (Table S4).

#### 3.2.3 Two pathways leading to the aerosol acidity difference

As aerosol pH is calculated as $\left[{log}_{10}(LWC)-{log}_{10}\left(H_{\text{air}}^{+}\right)-3\right]$, all mechanisms affecting aerosol pH must be through the modification of LWC, $H_{air}^{+}$, or both (LWC and $H_{air}^{+}$ are expressed as mass per unit volume of air, $\mu g\;m^{-3}$). We quantitatively separate the contributions of individual factors to the China–US pH difference into the LWC-modifying pathway and the $H_{\text{air}\;}^{+}$-modifying pathway (Fig. 6). To achieve this, we use the MTSM to quantify the contributions of individual factors to the differences in ${log}_{10}(LWC)$ and $\left[-{log}_{10}\left(H_{air}^{+}\right)-3\right]$ between the two countries, with the same approach as we did for pH (LWC and $H_{air}^{+}$ are two output variables directly predicted by ISORROPIA). The results show that the changes in both LWC and $H_{\text{air}\;}^{+}$ lead to increases in aerosol pH when conditions change from those in the USA to those in China.

Given that LWC increases with aerosol mass concentration (Song et al., 2019), higher component concentrations in China than in the USA increase LWC and, thus, increase aerosol pH (Fig. 6b). Through the LWC-modifying pathway, changes in ${SO}_{4}$, ${TNH}_{3}$, and ${TNO}_{3}$ lead to increases in pH (0.15–0.3) (Fig. 6b), which are consistent in all three groups. Compared to other groups, the observation group represents a higher pH increase due to Cl and a higher pH decrease due to RH (Fig. 6b), mainly because of the larger differences in Cl concentrations and RH for this group than for other groups (Table S4).

Through the $H_{air}^{+}$-modifying pathway, the effects of individual factors on pH changes vary (Fig. 6c). Increases in acidic components $\left({SO}_{4}\right.$ and $\left.{TNO}_{3}\right)$ increase $H_{\text{air}\;}^{+}$ and thus decrease aerosol pH (Fig. 6c). Increases in ${TNH}_{3}$, TCl, and NVCs, on the other hand, decrease $H_{\text{air}\;}^{+}$ and increase aerosol pH (Fig. 6c). By increasing $H_{\text{air}\;}^{+}$, increased ${SO}_{4}$ decreases pH by 0.7–1.2 units, showing a much stronger acidic capacity than another acidic component, ${TNO}_{3}$, which only decreases pH by 0.17–0.27 units (Fig. 6c). Compared to the USA, China is in a ${TNH}_{3}$-rich condition. The molar ratios of $\left[{TNH}_{3}\right]/\left(2\times \left[{SO}_{4}\right]+\left[{TNO}_{3}\right]+[TCl]\right)$ in China vs. in the USA are 3 vs. 1.4, 2.0 vs. 1.0, and 2.4 vs. 1.5 in the observation, non-weighted, and population-weighted groups, respectively. Changing the conditions from the USA to China, ${TNH}_{3}$ plays the most important role in neutralizing the acidic components and driving the pH increase in the $H_{air}^{+}$-modifying pathway (Fig. 6c).

For individual factors, the net changes in pH are a result of the combination of the two pathways. For example, increased ${SO}_{4}$ increases LWC and $H_{\text{air}\;}^{+}$ simultaneously. The increase in LWC increases aerosol pH, while the increase in $H_{\text{air}\;}^{+}$ decreases aerosol pH. All three groups suggest that the effect of $H_{air}^{+}$ on pH overwhelms that of LWC on pH, leading to a net decrease in pH from an ${SO}_{4}$ increase (Fig. 6). Increased ${TNH}_{3}$ increases pH in both pathways, adding up to a larger increase in pH (Fig. 6). Increased ${TNO}_{3}$ through these two pathways, however, is associated with opposite effects on pH which are comparable in magnitude and thus tend to offset each other (especially in the observation group) (Fig. 6). This explains the aforementioned small change in pH from the ${TNO}_{3}$ increases. Combining all the factors, both pathways increase aerosol pH (Fig. 6b and c), resulting in the large difference in aerosol acidity between these two countries (Fig. 6a).

To facilitate a follow-up sensitivity test to link the two pathways with mass concentration and chemical composition, we define the total mass concentration as the sum of the mass concentrations of all the eight input components (i.e., ${Na}^{+}$, ${SO}_{4}$, ${TNH}_{3}$, ${TNO}_{3}$, TCl, ${Ca}^{2+}$, $K^{+}$, and ${Mg}^{2+}$), including both gas and particle phases, and the chemical composition as the composition of the eight components in the aerosol (gas + particle) system. The observation group shows that the total mass concentration in China is 8.4 times that in the USA, and the chemical composition in China is richer in ${TNH}_{3}$ than that in the USA (as illustrated by the ratios of $\left[{TNH}_{3}\right]/\left(2\times \left[{SO}_{4}\right]+\left[{TNO}_{3}\right]+[TCl]\right)$ mentioned above). It has been found that both LWC and $H_{\text{air}\;}^{+}$ are affected by mass concentration and aerosol composition (Guo et al., 2015; Zheng et al., 2020; Xie et al., 2020). To investigate how the differences in mass concentration and composition between China and the USA are associated with the LWC- and $H_{air}^{+}$-modifying pathways and consequently the pH difference, we first increase the mass concentrations of individual input components in the US case by a constant factor of 8.4, whereby we obtain an intervening case representing the overall pollution level as in China but with the chemical composition feature as in the USA (Table S4, sensitivity test). From the intervening case, we then shift the composition of the US case to that of China (Table S4, sensitivity test). Note that throughout this sensitivity test, meteorological conditions are held constant. The first step, by increasing the mass concentration, yields an increase of 1.02 units in the aerosol pH, which is mainly achieved through the LWC-modifying pathway (1.06 units) instead of through the $H_{\text{air}\;}^{+}$-modifying pathway (–0.04 units) (Fig. S7a–c). The second step that changes the chemical composition shows a further increase of 0.76 units in the aerosol pH, which is mainly achieved through the $H_{air}^{+}$-modifying pathway (0.88 units). The LWC-modifying pathway plays a minor role (–0.11 units) in this step (Fig. S7d–f). This sensitivity test reveals that the LWC-modifying pathway is mainly associated with the change in mass concentration and the $H_{air}^{+}$-modifying pathway is mainly associated with the change in chemical composition.

It is surprising that in the first step, pH changed when the concentrations of all chemical components were scaled by a common factor. This means that pH changes with mass concentration of the aerosols (gas + particle) even though all chemical component mole fractions hold. Further investigation shows that increasing the aerosol concentration drives ${TNO}_{3}$ and ${TNH}_{3}$ partitioning toward particle phases $-\varepsilon \left({NH}_{4}^{+}\right)$ and $\varepsilon \left({NO}_{3}^{-}\right)$ increase from 0.4 and 0.6 to 0.6 and 0.98, respectively. Given the weak acidity of ${NO}_{3}^{-}$, the particle is ultimately neutralized by the increased ${NH}_{4}^{+}$. The repartitioning in response to the increase in mass concentration is thus key to the pH shift and can be explained by Henry’s law; i.e., $\left[A_{aq}\right]=H_{A\cdot p_{A}}$, where $\left[A_{aq}\right]$ is the aqueous-phase concentration of component A in units of moles per liter of water, $p_{A}$ is the partial pressure of A in the gas phase, and $H_{A}$ is Henry’s law coefficient (Seinfeld and Pandis, 2006). $\left[A_{aq}\right]$ is proportional to $c_{A}/LWC\;(c_{A}$ denotes the particle-phase concentration of A; note that LWC and $c_{A}$ are expressed in mass per unit volume of air and $\left[A_{aq}\right]$ is expressed in moles per unit volume of water). Increasing the concentrations of all chemical components by a common factor increases $p_{A}$ (due to the increase in the gas-phase concentration of A) but does not change $\left[A_{aq}\right]$ (because both $c_{A}$ and LWC increases in the same direction by the same magnitude). According to Henry’s law, more gas-phase A will thus shift toward the particle phase to achieve a thermodynamic equilibrium of the new system.

We find that by increasing the concentration of every component by a constant factor, the magnitude and direction of the resulting change in pH are sensitive to the fraction of ${TNH}_{3}$ in the aerosol system while insensitive to the ratio of ${SO}_{4}$ to ${TNO}_{3}$. Based on an ${NH}_{4}^{+}-{SO}_{4}^{2-}-{NO}_{3}^{-}-H_{2}O$ system, we conduct a series of sensitivity tests to investigate the change in aerosol pH in response to the multiplication of a constant factor of 8.4 (Fig. S8). The change in pH reduces gradually from 1.2 units to 0.8 units when the ${TNH}_{3}$ mass fraction of the system decreases from 67 % to 27 % (Fig. S8). With further decreases in the ${TNH}_{3}$ fraction, the increase in pH diminishes rapidly, becomes negative when the ${TNH}_{3}$ mass fraction is lower than 25 %, and is −0.6 when the ${TNH}_{3}$ mass fraction is 17 % (Fig. S8). Under a constant ${TNH}_{3}$ mass fraction, the change in pH remains generally constant across a wide range of the mass ratios of ${SO}_{4}$ to ${TNO}_{3}$ (from 5:1 to 1:5) (Fig. S8). In populated continental regions, mass fractions of ${TNH}_{3}$ are often high (Bencs et al., 2008; Behera and Sharma, 2010; Zheng et al., 2015; Cheng et al., 2016; Guo et al., 2017b), and an increase in mass concentration thus typically increases the aerosol pH.

Such an assessment by tracking pathway- and step-specific contributions provides a better understanding of the pH difference between China and the USA. We show that through the LWC-modifying pathway, the increases in aerosol components consistently lead to increases in pH and that through the $H_{air}^{+}$-modifying pathway, the effects of different components on pH vary in direction. If the LWC-modifying pathway dominated the pH changes over the $H_{air}^{+}$-modifying pathway, aerosol mass concentrations would be the main factor driving the aerosol acidity difference between China and the USA and one could simply attribute the difference in aerosol acidity to the fact that China is more polluted than the USA. In contrast, if the $H_{air}^{+}$-modifying pathway dominated, chemical composition would be the dominant factor and the compound profiles of precursors emissions, which affect the fractions of the corresponding aerosol components in the air, would play an important role. While there has been debate about whether mass concentration or chemical composition plays a more important role in determining aerosol pH (Cheng et al., 2016; Guo et al., 2017a; Pye et al., 2020; Zheng et al., 2020), our results suggest that both are important in explaining the China–US pH difference (Fig. 6b and c). The three groups are not consistent with each other in which pathway contributes more than the other to the pH difference, but they all suggest that the two pathways are comparable in terms of their effects on aerosol pH (Fig. 6b and c).

Our results, showing the importance of both mass concentrations associated with LWC and chemical composition associated with $H_{\text{air}}^{+}$ and a minor role of temperature, seem in some aspects to contradict a previous study (Zheng et al., 2020) which highlighted LWC and temperature instead of chemical composition as the most important factors explaining the pH difference between China (NCP) and the USA. We note that the difference in the conclusions is reasonable when considering the differences in the specific cases examined in these two studies. The previous study compared the conditions in NCP in winter with those in the southeastern USA in summer (SE USA). Because of the differences in latitude (north for China vs. south for the USA) and season (winter for China vs. summer for the USA), the difference in temperature between their scenarios (29 K) was an order of magnitude greater than those in our study, which has greater spatial and temporal coverage (2.6 K in the observation group, 5 K in the non-weighted group, and −1.4 K in the population-weighted group). Using the MTSM, we evaluate the pH difference between the NCP and SE-USA scenarios considered in the previous study. The results show that temperature accounts for 1.3 units of difference in aerosol pH between their two cenarios (Fig. S9), in line with what was previously reported (1.6 units).

In addition, ISORROPIA simulations show a LWC difference of $8.2\;\mu g\;m^{-3}$ between China (NCP) and the contiguous USA in the observation group in our study and $340\;\mu g\;m^{-3}$ between the scenarios considered in the previous study. The much larger LWC difference in the previous study compared to ours is mainly driven by the differences in pollutant concentrations. For example, the ${SO}_{4}$ concentration is as high as $156\;\mu g{\;m}^{-3}$ in the NCP scenario in the previous study but only $9.2\;\mu g{\;m}^{-3}$ in our study. Such differences in concentrations are reasonable, given that the previous study selected a severe haze event occurring in Beijing in winter 2013 as the scenario for China (NCP), while we use annual average levels over the NCP in 2017 as our case for China (NCP). Note that winter 2013 was a period when air pollution reportedly reached record high levels across northern China (Y. Wang et al., 2014; Li et al., 2016). Since 2013, China has launched strict controls on air pollutant emissions, and PM_2.5_ levels decreased significantly between 2013 and 2017 (Zhang et al., 2019). Therefore, the NCP scenario in the previous study should be more representative of short-term haze events in the pre-2013 period, while our China (NCP) case should be more representative of annual average levels in recent years.

## 4 Conclusion and implications

Based on extended ground-level measurements and regional air quality model simulations, we find significant differences in aerosol pH between China and the USA. Aerosols in the USA are on average more acidic with pH generally 1–2 units lower than in China. We propose an MTSM to identify the key factors leading to the pH difference. The MTSM analysis reveals the important role of ${TNH}_{3}$ in causing the pH difference and an opposing effect from ${SO}_{4}$, which partially offsets the positive effect of ${TNH}_{3}$ on the pH change. Other factors play relatively minor roles. Further investigation highlights two pathways related to the pH difference, one associated with changes in LWC and the other with changes in $H_{\text{air}}^{+}$. The increased mass concentration in China, compared to the USA, enhances LWC, and the change in chemical composition toward a ${TNH}_{3}$-rich condition reduces $H_{air}^{+}$. Both pathways facilitate the increases in aerosol pH in China and are comparable in terms of driving the pH increase.

Previous studies have suggested that low aerosol pH is associated with increased toxicity because of the increased solubility of transition metals in aerosol LWC, which induce airway injury and inflammation through the production of reactive oxygen species in vivo (Kim et al., 2015). The lower aerosol pH in the USA than in China implies that aerosols in the USA may be more toxic than in China. However, this implication should be interpreted with caution because there are other known pathways through which particulate matter can harm humans and the mechanisms of how particulate matter affects health are not completely understood (Armstrong et al., 2004). More studies are needed to assess the health outcomes associated with the disparity in aerosol pH between the two countries.

## Supplementary Material

supplement zhang aerosol acidity

## Figures and Tables

**Table 1.** Summary of the 1-year average values of mass concentration of water-soluble ions (WSI), gaseous and aerosol species, aerosol pH and meteorological parameters (as average ± standard deviation) in China and the United States during the study periods (i.e., 2017 for China and 2011 for the United States).

	China (*n* = 1845)	USA (*n* = 1191)

WSI ($\mu g\;m^{-3}$)	34.4 ± 25.5	5.7 ± 2.2
Temperature (K)	284.8 ± 11.7	287.4 ± 10.0
RH (%)	45.1 ± 17.6	71.4 ± 20.9
pH	4.3 ± 1.2	2.6 ± 0.7

Particle phase ($\mu g\;m^{-3}$)		

${SO}_{4}^{2-}$	9.2 ± 7.1	2.2 ± 1.3
${SO}_{3}^{-}$	12.1 ± 11.1	0.8 ± 0.9

${NH}_{4}^{2+}$	8.9 ± 8.0	0.8 ± 0.5
${\text{Cl}}^{-}$	2.2 ± 2.3	0.4 ± 0.1
${\text{Na}}^{+}$	0.7 ± 1.0	0.2 ± 0.2
${\text{K}}^{+}$	0.7 ± 0.6	0.1 ± 0.1

${\text{Ca}}^{2+}$	1.0 ± 0.1	0.3 ± 0.2
${\text{Mg}}^{2+}$	0.2 ± 0.1	0.1 ± 0.1

Gaseous phase ($\mu g\;m^{-3}$)		

${\text{NH}}_{3}$	18.0 ± 12.6	1.1 ± 1.7
HCl	1.9 ± 3.4	–
${\text{HNO}}_{3}$	1.0 ± 1.1	1.0 ± 0.6

Total ($\mu g\;m^{-3}$)		

${\text{TNH}}_{3}$	26.5 ± 17.2	1.9 ± 1.8
TCl	4.1 ± 4.5	–
${\text{TNO}}_{3}$	13.1 ± 11.2	1.8 ± 1.1

## Data Availability

The data presented in this paper and the observational data in China can be obtained from the corresponding author upon request. The observational data in China can also be obtained from the data-sharing platform by the Comprehensive Observation Network for Air Pollution in Beijing-Tianjin-Hebei and Its Surrounding Areas (http://123.127.175.60:8765/siteui/index, last access: 18 November 2019, China National Environmental Monitoring Centre, 2019). The observational data in the USA can be obtained from the Clean Air Status and Trends Network (CASTNET) (https://www.epa.gov/castnet, last access: 23 January 2021, United States Environmental Protection Agency, 2021) and Ammonia Monitoring Network (AMoN) (http://nadp.slh.wisc.edu/amon/, last access: 23 January 2021, National Atmospheric Deposition Program, 2021).
