# Supplementary material for: Significant contrasts in aerosol acidity between China and the United States
Source: Atmos Chem Phys. Author manuscript; Available in PMC 2023 Dec 15. (PMC10723067; doi:10.5194/acp-21-8341-2021)
Supplement: supplement zhang aerosol acidity [file NIHMS1893565-supplement-supplement_zhang_aerosol_acidity.pdf]

Supplement of Atmos. Chem. Phys., 21, 8341–8356, 2021
https://doi.org/10.5194/acp-21-8341-2021-supplement
© Author(s) 2021. CC BY 4.0 License.

Atmospheric Chemistry and Physics
Open Access

Supplement of

Significant contrasts in aerosol acidity between China and the United States

Bingqing Zhang et al.

Correspondence to: Huizhong Shen (shenhz@sustech.edu.cn)

The copyright of individual parts of the supplement might differ from the article licence.

Supplementary Information

Text. S1 Effects of ammonium on aerosol pH

The result of MTSM indicates that the difference in TNH_3 is one of the predominant reasons causing the pH difference. In order to study the effect of TNH_3, we conduct sensitivity tests for China and the US separately to investigate the responses of aerosol pH to changing TNH_3. We change the TNH_3 concentrations from 0.1 to 1000 μg m^{-3} while keep all other components constant at their annual average levels based on observation data (Table 2). We also use simulation data with population as the weight to study the effects, which consider other areas in China where TNH_3 concentration is not as high as in NCP. The results are shown in Fig. S10. It is clearly illustrated that, over a large range of TNH_3 concentrations, aerosol pH increases with the increase in TNH_3 because the production process of NH_4^+ from NH_3 consumes aqueous H^+. The local sensitivity of pH to TNH_3, expressed as the pH increase per tenfold increase in TNH_3 at current TNH_3 level, is higher in the US (3.0 based on observational data and 1.6 based on simulation data) than in China (0.4 based on observational data and 1.2 based on simulation data), indicated a higher sensitivity of aerosol pH to TNH_3 in the US than in China. Besides, we find that the responses of pH to TNH_3 are nonlinear and anisotropic. With all others equal, pH in the US could be closer to the level in China if the TNH_3 increases to the level in China. On the other hand, the pH in China would be lower than the US if the TNH_3 decreases to the US level because of the relative higher abundances of acidic components (SO_4, TNO_3, TCl) than basic ions (TNH_3, NVCs) (Fig. S10a). In both countries, the sensitivities would quickly diverge from the original values toward higher values as TNH_3 decreases, with the sensitivities in China changing at a faster pace. As TNH_3 increases, however, the sensitivities in these two countries would gradually become constant, stabilizing at comparable levels (0.002 pH unit per TNH_3 increase in both two countries). Results based on simulation data are similar with results based on observational data, especially the sensitivity of aerosol pH at high level of TNH_3 (represented by similar slope). Higher pH values in China based on simulation data at low TNH_3 level (1-10 μg m^{-3}) could be caused by lower SO_4^{2-} concentrations. However, lower value of aerosol pH at high level of TNH_3 (> 50 μg m^{-3}) based on simulation data even with lower SO_4^{2-} concentrations indicates the limit effect of TNH_3 at this level and potential effect of other components.

The effects of TNH_3 on the gas-particle partitioning of NH_3-NH_4^+ and HNO_3-NO_3^- are illustrated in Fig. S10b and S10c, showing a decreasing trend of $\varepsilon(NH_4^+)$ and an increasing trend of $\varepsilon(NO_3^-)$ as TNH_3 increases. In the range of observation cases the value of $\varepsilon(NH_4^+)$ in China is smaller than in the US, suggesting excess presence of TNH_3 compared to other aerosol components (e.g., TNO_3 and SO_4^{2-}). The gas to particle partitioning of NH_3 produces inorganic ammonium salt of ammonium bisulfate (NH_4HSO_4) and ammonium sulfate ($(NH_4)_2SO_4$) first because the affinity of sulfuric acid for NH_3 is much larger than that of nitric and hydrochloric acid for NH_3, especially when TNH_3 concentration is relatively low (Behera et al., 2013); The excess TNH_3 may also react with nitric acid and hydrochloric acid to form salt of NH_4NO_3 and NH_4Cl which will dissolve in the aerosol liquid water (Zhao et al., 2016). That is why the increase of $\varepsilon(NO_3^-)$ is small at the beginning and gradually become faster later. At the same level of TNH_3, $\varepsilon(NH_4^+)$ in China is higher than $\varepsilon(NH_4^+)$ in the US,

causing by formation of NH_4NO_3 due to higher level of TNO_3. Both the lower $\varepsilon(NH_4^+)$ and higher $\varepsilon(NO_3^-)$ in China estimated by the sensitivity curves are consistent with observations.

Therefore, the ratio of $[NH_4^+]$ to different acid ions ($[SO_4^{2-}]$, $[NO_3^-]$, $[Cl^-]$) can be used to indicate the relative abundance of ammonia. To further investigate the different levels of abundance of ammonia, we tend to look at groups of real cases, instead of the average values only. We divide the observation data into three groups based on neutralization condition of particle phase NH_4^+. Group A contains the observations when $[NH_4^+] < 2\times[SO_4^{2-}]$, when available NH_4^+ cannot completely balance aerosol SO_4. Group B consists of the data points when $2\times[SO_4^{2-}] < [NH_4^+] < 2\times[SO_4^{2-}]+[NO_3^-]+[Cl^-]$ when most of the aerosol SO_4 is in the form of SO_4^{2-} and excess $[NH_4^+]$ is available to stabilize nitrate and chloride driving the gas phase to shift to the particle phase. Group C contains the data points when $[NH_4^+] > 2\times[SO_4^{2-}] +[NO_3^-] +[Cl^-]$, where available NH_4^+ is enough to balance particle phase anions. The distribution of pH values in three groups in two countries is shown in Figure S11 by boxplots. Note that no data in the US fall in Group C, making up only two groups in Figure S11 (b). Overall, cases in groups B and C with higher relative abundance of ammonia are more likely to have higher aerosol pH than in group A, though the relationship is not linear and does not happen in all the cases. Note that although the relative abundance of NH_4^+ in group B is smaller than in group C, the transition from group B to group C due to TNH_3 increase does not always happen. Because if TNH_3 increase in an aerosol system with $2\times[SO_4^{2-}] < [NH_4^+] < 2\times[SO_4^{2-}]+[NO_3^-]+[Cl^-]$, $[NH_4^+]$ would increase, and more TNO_3 and TCl would shift into the particle phase, leading to the increase of WSI concentration. However, the average WSI concentration in group B is 55.03±46.79 μg m^{-3} in China, significantly higher than that in group C in China (31.60±20.29 μg m^{-3}). LWC in group B (22.90±7.38 μg m^{-3}) is also higher than that in group C (14.37±16.85 μg m^{-3}). We find that most of the cases in group B could be identified as highly polluted cases where large amount of NH_4NO_3 is formed and dissolves in the aerosol water.

Throughout the observed cases, 85% in China are in Group C (i.e., aerosol systems with excess NH_4^+), and 55% in the US are in Group A (i.e., aerosol systems with insufficient NH_4^+). Overall, the positive sensitivity of pH to TNH_3 and the different dominant groups in these two countries (Group C in China, Group A in the US) suggest that the high abundance of TNH_3 in China increases the aerosol pH and is one of the major reasons for the pH difference between the two countries.

Text. S2 The relationship between sulfate/nitrate and aerosol pH

Besides the effect of TNH_3 discussed in Text. S1, other species, especially the acidic species which mainly include SO_4 and TNO_3, could also affect aerosol pH because of their effects on H^+_{air} concentration as well as on LWC (Ding et al., 2019). This effect is investigated in a sensitivity test by changing TNO_3 or SO_4 concentration while keeping other inputs constant as

the average levels (Fig. S12). Similar to the MSTM results as shown in Fig. 6, elevated SO_4^{2-} significantly increases aerosol acidity by increasing H^+_{air}. On the other hand, elevated TNO_3 only slightly increases H^+_{air}, indicating a weaker acidity than that of SO_4^{2-}, in line with the result in a previous study (Guo et al., 2017b). This is partially due to the semi-volatile property of TNO_3 (Ding et al., 2019). Notably, even in China where $\varepsilon(NO_3^-)$ are mostly close to 1, the variation of aerosol pH with TNO_3 (roughly equals to NO_3^- in this case) is also subtle. Therefore, for two systems with different moles of SO_4^{2-} and NO_3^- neutralized by same moles of NH_4^+, the system with more SO_4^{2-} will likely have a lower pH. This result indicates that higher aerosol acidity is associated with higher availability of SO_4^{2-} rather than TNO_3, which can be confirmed by observed data in Fig. S13.

Based on observation data, 74.5% of the cases in China have NO_3^-/SO_4^{2-} molar ratio larger than one, while only 22.3% in the United States. The different NO_3^-/SO_4^{2-} ratios, could subsequently affect other aerosol properties, such as aerosol water uptake ability, which is one of the important reasons causing haze events in China during wintertime (Xie et al., 2019; Wang et al., 2020). Although nitrate aerosol and sulfate aerosol absorb similar amounts of water per mass, heavy haze events in China are usually associated with increased LWC with enhanced RH levels under nitrate-dominate condition (Wang et al., 2020).

In order to study this effect, we categorize the observation data into a nitrate-rich group (Group N, where $[NO_3^-]/[SO_4^{2-}] > 3$) and a sulfate-rich group (Group S, where $[NO_3^-]/[SO_4^{2-}] < 1$) and compare these two groups under different RH conditions. The ratio 3 in group N is mentioned in lab studies and is a more typical value of nitrate-rich conditions in field observations (Ge et al., 1998; Xie et al., 2020).

The results in Fig. S14 show that aerosol pH values in the same groups in China and the US have similar responses to the changes in RH. In both countries, as RH increases, the pH in group N decreases, and the pH in group S increases (Fig. S14a). Both the values and the increasing rate of LWC in group N is larger than in group S, suggesting a higher water uptake ability in nitrate-rich condition, which is likely due to higher aerosol mass compared with group S as shown in Fig. S14f. The nearly two times aerosol mass in group N as in group S indicates the co-condensation effect of nitrate aerosol and LWC (Guo et al., 2017a), which suggests that NO_3^- formed in aerosol leads to a higher LWC due to the increase in aerosol mass, while higher LWC dilutes H^+_{air} and increases pH, which is favorable for more HNO_3 shifting from gas phase to particle phase and thus continually increases particle NO_3^- concentration. This effect will reach a balance when most of the gas phase HNO_3 is in the particle phase with enough NH_4^+, and, therefore, $\varepsilon(NO_3^-)$ is close to 100% in group N in the two countries (Fig. S14e). Besides, water uptake by hygroscopic aerosols increases the aerosol surface area and volume, enhancing the hydrolysis of N_2O_5 across particles and forming NO_3^- (Tian et al., 2018; Wang et al., 2020).

The condition in group N usually has a higher LWC and aerosol mass, due to the mutual promotion between LWC and particle nitrate. And such a condition in group N occurs more often in China than in the United States, which is probably one of the reasons leading to high particle concentrations on hazy days in China.

The nitrate/sulfate ratio depends on the emission ratio of NO_x/SO_2, the availability of cations due to the dependency of $\varepsilon(NO_3^-)$ on TNH_3 (Fig. S10c), and other factors such as the atmospheric oxidizing capacity. Further investigation into the total emissions shows that the emission molar ratios of $[NO_x]/[SO_2]$ are close to 3:1 in both countries (2.92 In China in 2017 and 3.12 in the US in 2011 when assuming the emission NOx is in the form of NO_2), indicating that the emission difference is not the major factor leading to the nitrate/sulfate ratio difference. On the other hand, the emission molar ratio of $[NH_3]/([NO_x]+2\times[SO_2])$ in China (0.75) is 1.6 times higher than that in the US (0.46), which is consistent with the measured high relative abundance of TNH_3 in China and confirms that high availability of cations (mainly NH_4^+ caused by high NH_3 emission) is one of the causes for the high nitrate/sulfate ratio in China.

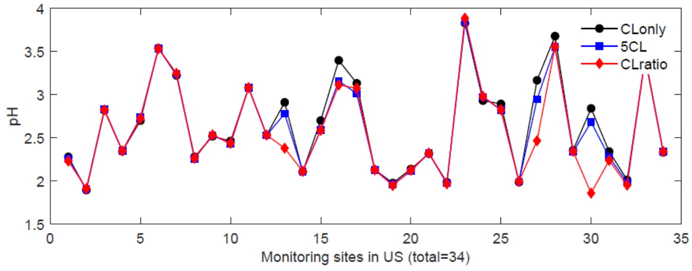

Fig. S1 Comparison of results in pH calculation when using different methods to estimate HCl concentration in the United States. Group “Cl only” means using particle Cl^- concentration as total Cl and ignore gas phase HCl; group “5Cl” means assuming total Cl equals to 5 times particle Cl^- concentration therefore HCl concentration equals to 4 times particle Cl^- concentration; group “Cl ratio” means using measured particle Cl^- concentration divided by CMAQ simulation partitioning ratio to estimate the amount of total Cl. The result showed the three methods will lead to essentially the same pH at most of the monitoring sites.

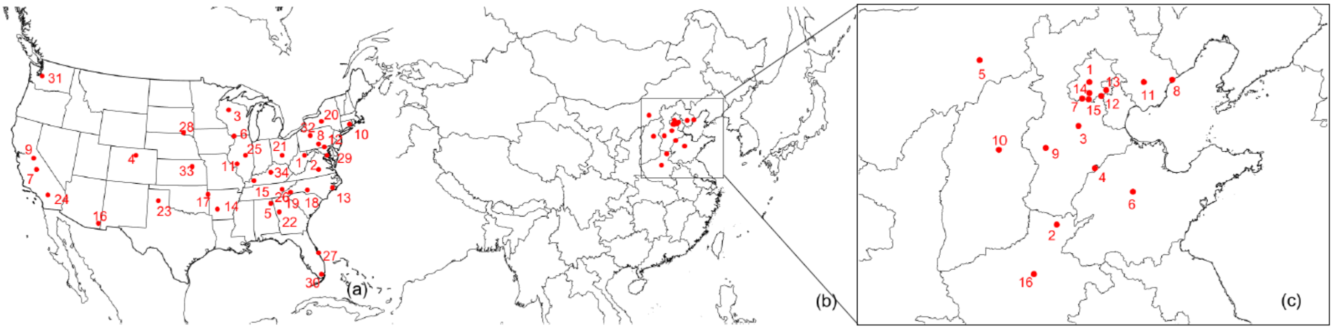

Fig. S2 Location of monitoring sites used in this study in the United States(a) and China(b), with a close-up North China Plain (c). The world shapefiles were obtained from Esri (ArcGIS Hub, Countries WGS84. June 21, 2019. http://www.arcgis.com/home/item.html?id=30e5fe3149c34df1ba922e6f5bbf808f)

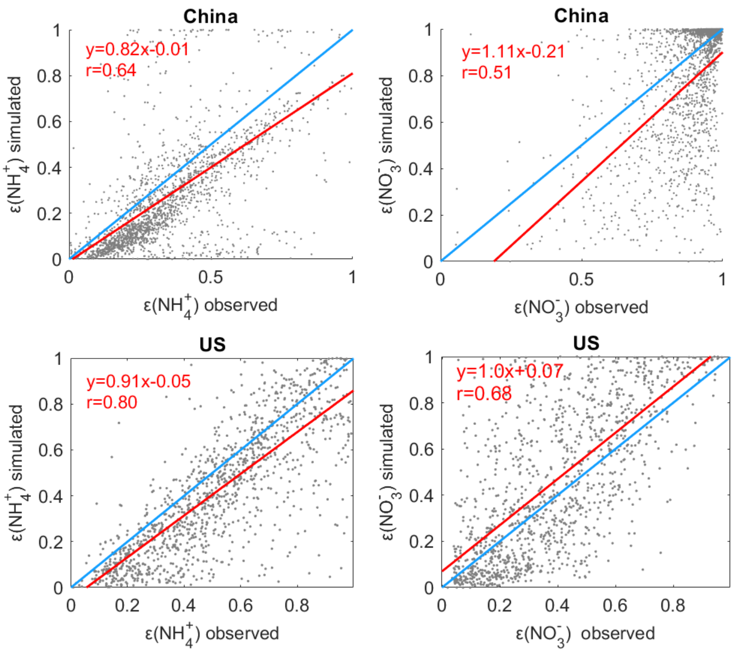

Fig. S3 Observed ε(NH_4^+) (left column) and ε(NO_3^-) (right column) in China (top row) and the United States (bottom row) versus simulated by ISORROPIA-II. The regression line(red), 1:1 line(blue), and the regression equation and correlation coefficient r are shown on each panel.

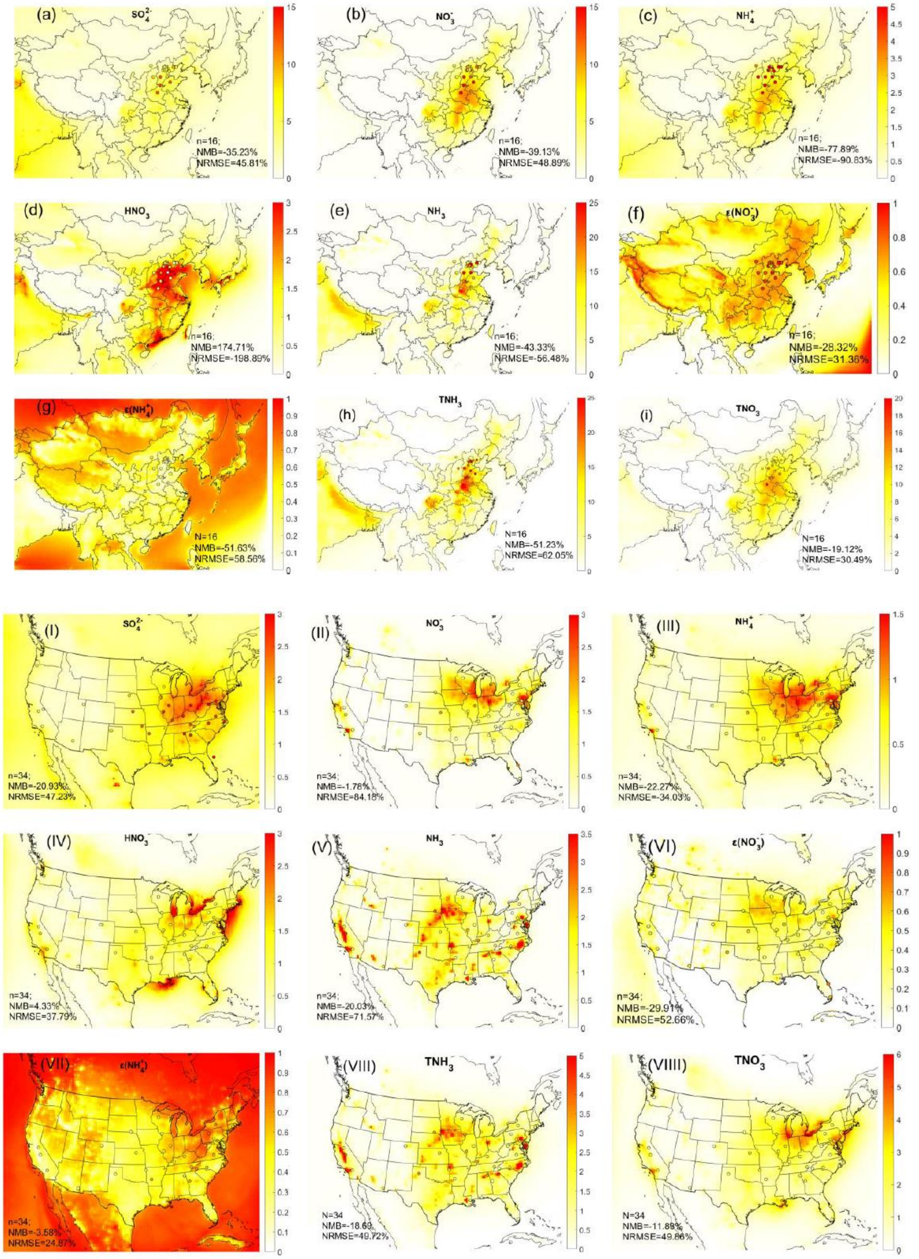

Fig. S4 Annual mean concentrations of $PM_{2.5}$ components SO_4^{2-}, NO_3^-, NH_4^+, gaseous components HNO_3 and NH_3 and the partitioning including ε(NO_3^-) and ε(NH_4^+) based on CMAQ simulations (colored map) and observations (colored dots) in China (panels a-g) and in the United States (panels i-vii). Normalized mean bias (NMB) and normalized root mean square error (NRMSE) are shown on each panel. The world shapefiles were obtained from Esri (ArcGIS Hub, Countries WGS84. June 21, 2019. http://www.arcgis.com/home/item.html?id=30e5fe3149c34df1ba922e6f5bbf808f)

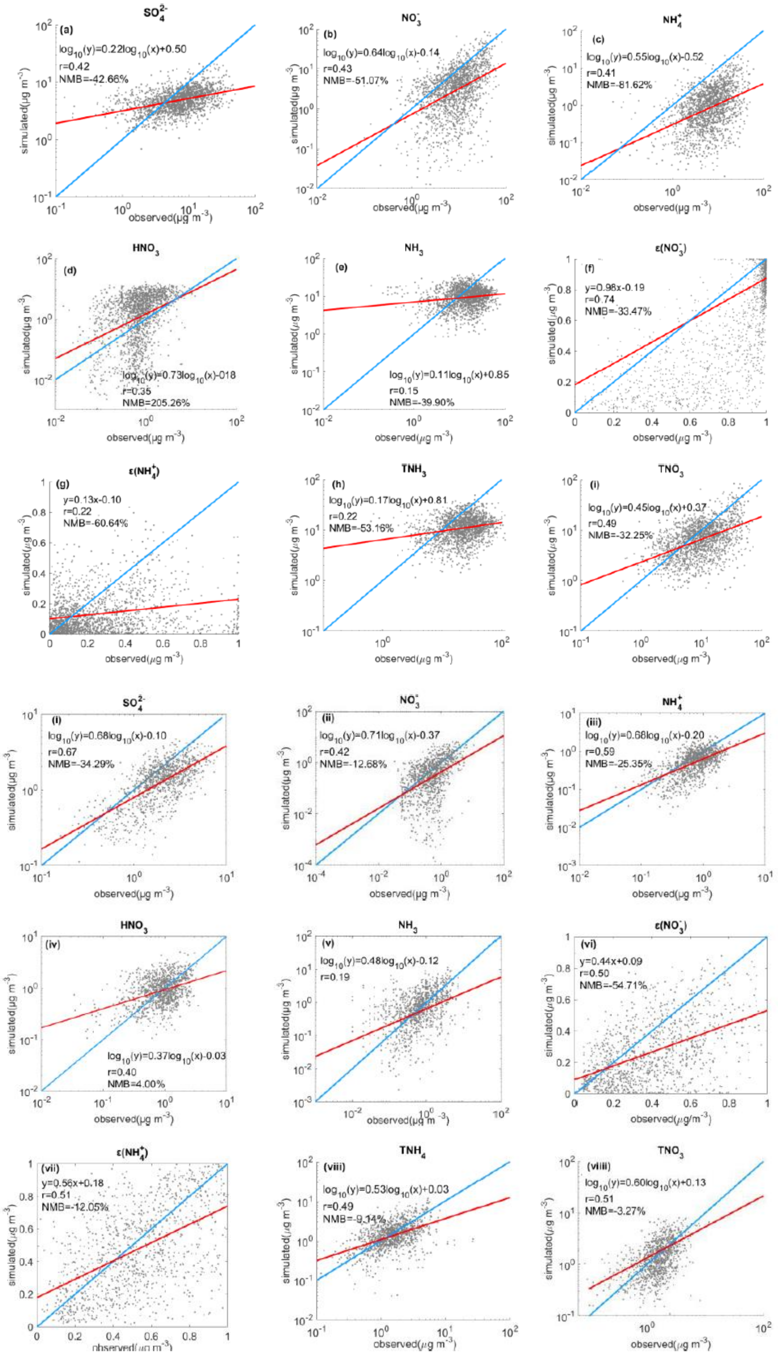

Fig. S5 Comparison of daily observed and CMAQ simulated aerosol component concentrations of SO_4^{2-}, NO_3^-, NH_4^+, gaseous concentrations of HNO_3 and NH_3 and the partitioning ε(NO_3^-) and ε(NH_4^+) in China (panels a-g) and in the United States (panels i-vii). The regression line (red), 1:1 line (blue), regression equation and correlation coefficient r are shown in each panel.

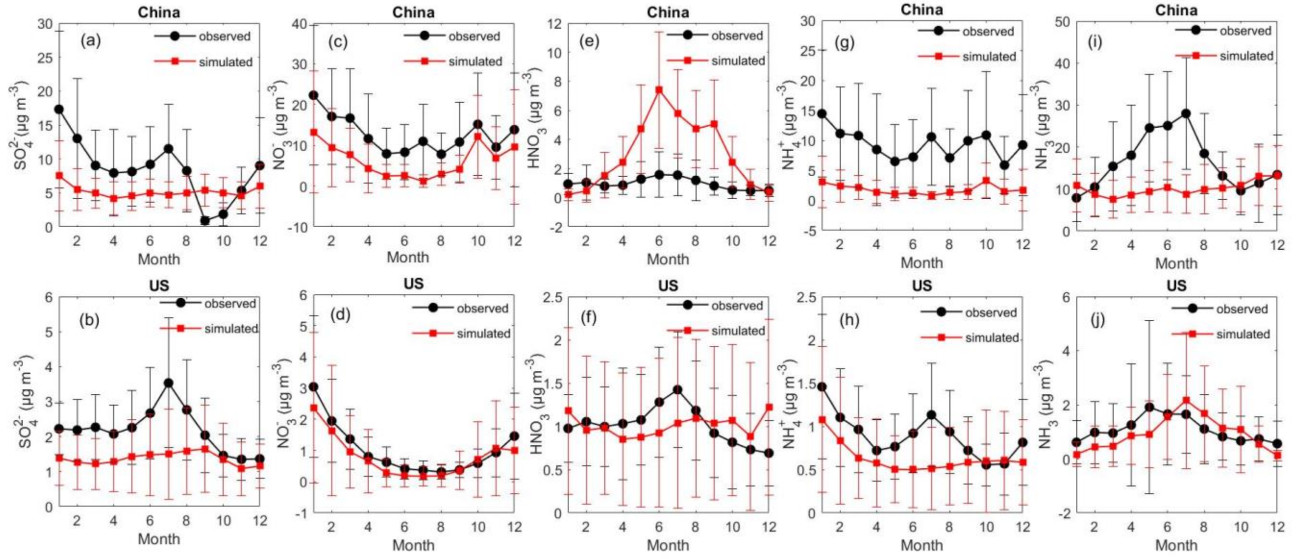

Fig. S6 Monthly average concentrations of SO_4^{2-}, NO_3^-, HNO_3^-, NH_4^+, NH_3: observed versus CMAQ simulated data in China (panels a, c, e, g, i) and in the United States (panels b, d, f, h, j). The error bars represent the standard deviation of all the cases in each month.

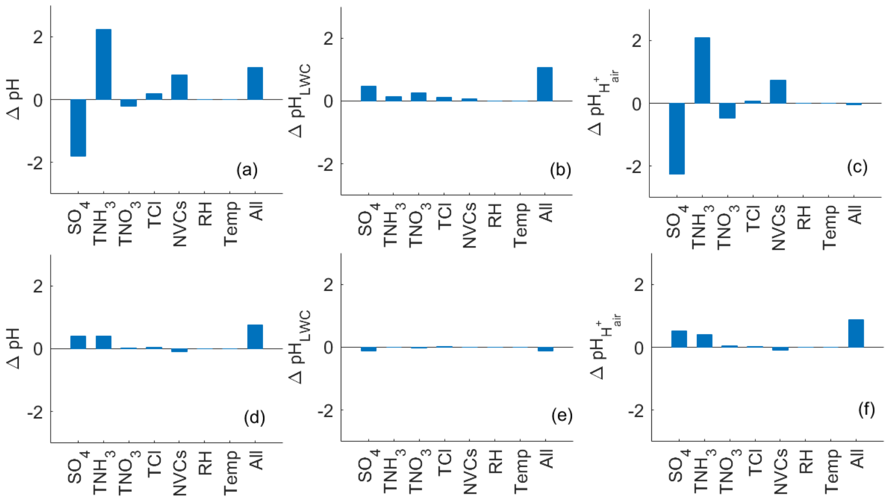

Fig. S7 Step-specific contributions of individual factors to the pH difference between China and the US. (a), (b), and (c) show the contributions of individual components and meteorological factors to (a) total difference of aerosol pH (ΔpH), (b) through the pathway of LWC (ΔpH_{LWC}), (c) through the pathway of H^+_{air} ($\Delta pH_{H^+_{air}}$) between the US case and an intervening case with the concentrations of all components in the US case multiplied by a constant factor of 8.4. The former is chosen as the starting point, and the latter is chosen as the ending point. (d), (e), and (f) show the contributions of individual components and meteorological factors to (d) total difference of aerosol pH (ΔpH), (e) through the pathway of LWC (ΔpH_{LWC}), (f) through the pathway of H^+_{air} ($\Delta pH_{H^+_{air}}$) between the intervening case and the China case. The former is chosen as the starting point, and the latter is chosen as the ending point. The inputs are shown in Table. S4, sensitivity test.

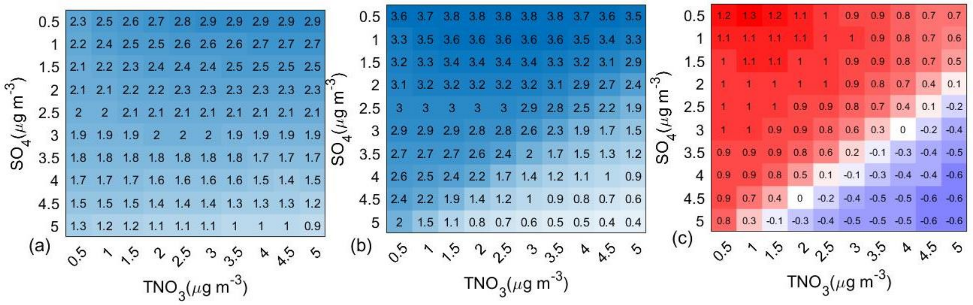

Fig. S8 Sensitivity tests showing the pH changes in response to different levels of SO_4 and TNO_3 in an NH_4^+-SO_4^{2-}-NO_3^--H_2O system. (a) The pH of an aerosol with fixed TNH_3 (2μg m^{-3}) and varied SO_4 and TNO_3 (from 0.5 μg m^{-3} to 5 μg m^{-3}) (b) The pH after multiplying all the inputs in (a) by a factor of 8.4. Note that the SO_4 and TNO_3 levels shown along the axes are the initial levels before multiplication. (c) pH differences between (b) and (a) (b minus a).

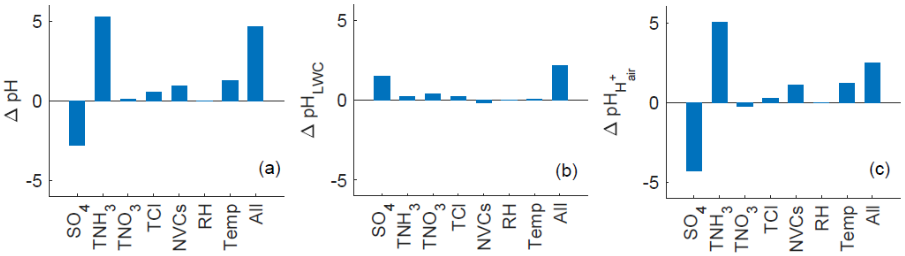

Fig. S9 Contributions of individual components and meteorological factors to (a) total difference of aerosol pH (ΔpH), (b) through the pathway of LWC (ΔpH_{LWC}), (c) through the pathway of H^+_{air} ($\Delta pH_{H^+_{air}}$) calculated by Multivariable Taylor Series Method (MTSM) between the NCP scenario and the US-SE scenario in Zheng's study (Zheng et al., 2020). For individual factors, the sum of the contributions through the two pathways yields the net contribution of this factor to aerosol pH. The case in the United States is chosen as the starting point, and China as the ending point.

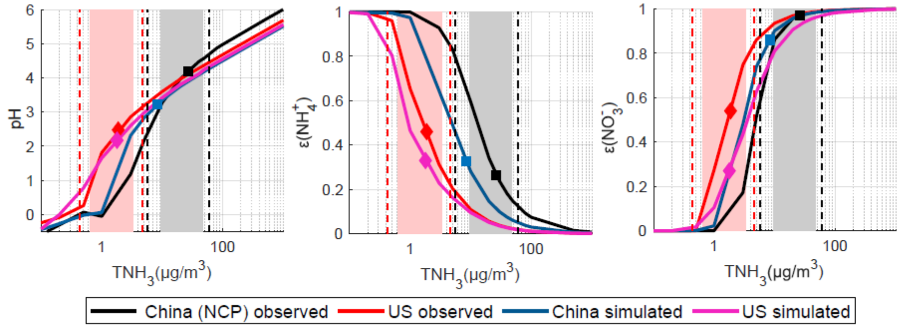

Fig. S10 Responses of pH, ε(NH_4^+) and ε(NO_3^-) to the change of TNH_3 from 0.1 to 1000 μg m^{-3} while keep all other components constant at their annual average levels. The shaded areas show the TNH_3 concentration ranges that covers 75% of the observed cases in the countries, the dashed lines show the 5th and 95th percentiles of the observed cases, the black square and the red diamond mark the average TNH_3 levels in China and the United States, respectively.

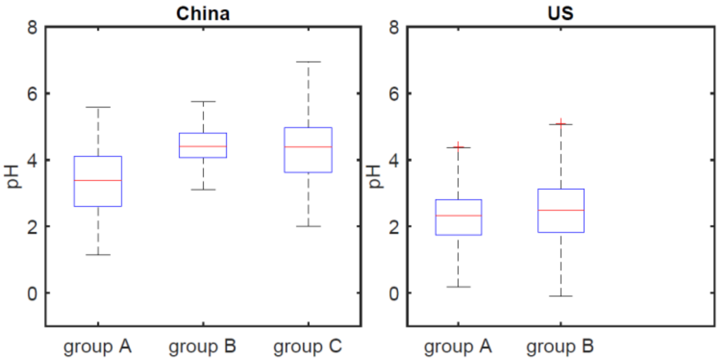

Fig. S11 Distribution of aerosol pH in three groups with different relative abundance of ammonia in two countries. Outliers recognized by data out of the range of three times median absolute deviations from the median are removed.

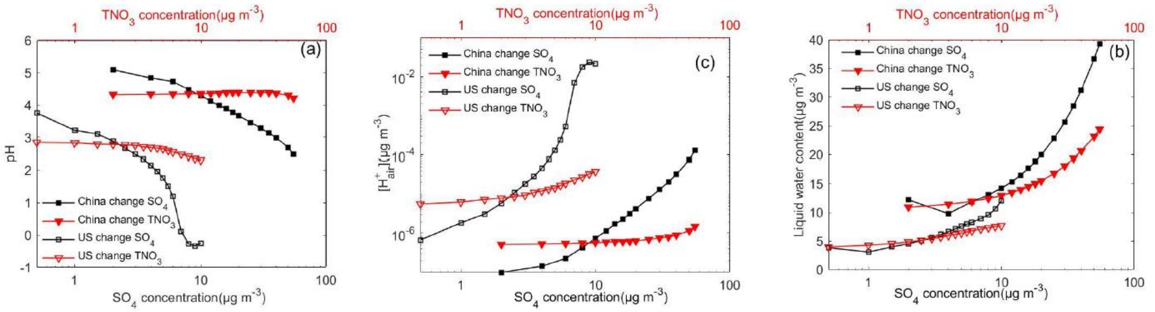

Fig. S12 Values of pH, liquid water content and H^{+}_{air} to the change of TSO_4 and TNO_3 concentration in China and the United States.

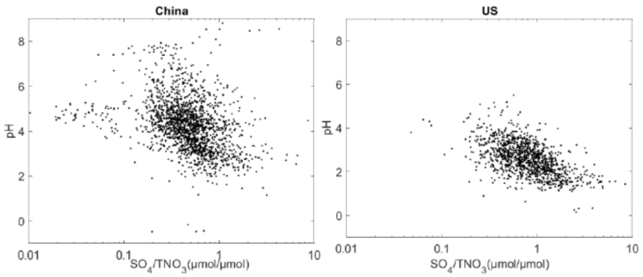

Fig. S13 Relation between aerosol pH and TSO_4/TNO_3 molar ratio in China (left) and the United States (right) based on observational data.

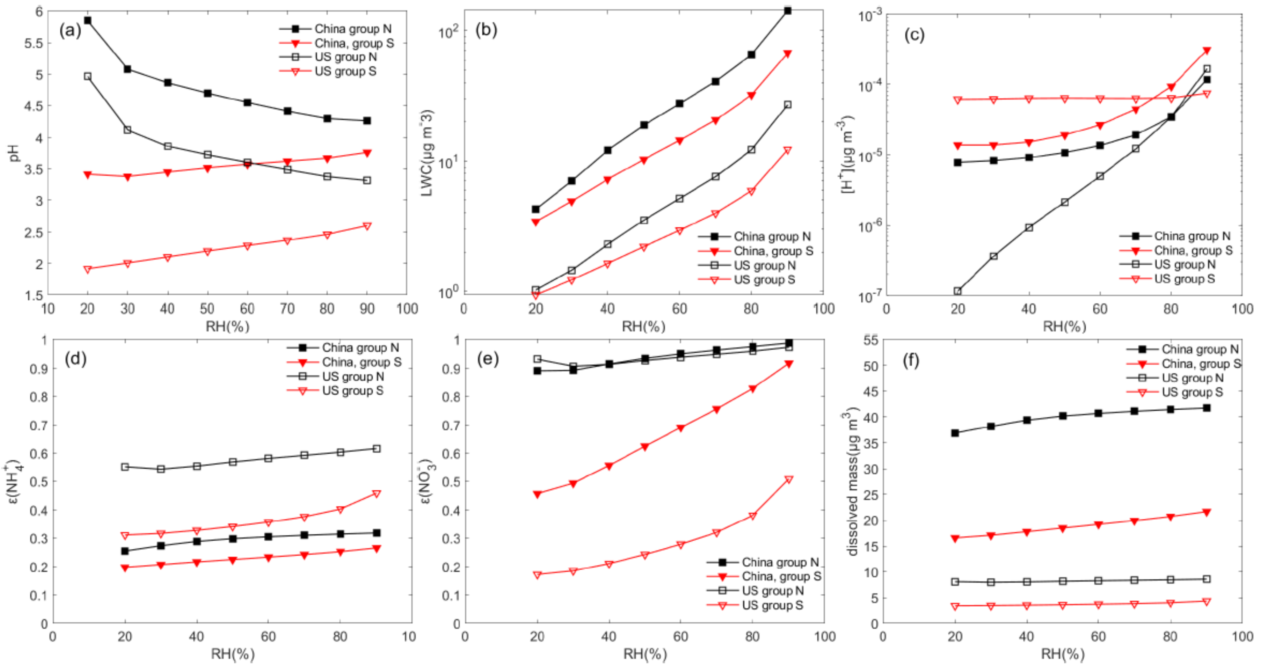

Fig. S14 Values of pH, LWC, H^+_{air}, ε(NH_4^+), ε(NO_3^-) and dissolved mass in group N and group S under different RH conditions in China and the United States. China: group N, n=410; group S, n=470; US: group N, n=72; group S, n=1119.

Table S1. List of the 16 monitoring sites in China

Monitoring Sites in China			
**No.**	Site name	Latitude	Longitude
**1**	Chinese Research Academy of Environmental Science（CRAES)	40.04	116.42
**2**	Anyang	36.09	114.39
**3**	Baoding	38.87	115.52
**4**	Dezhou	37.45	116.32
**5**	Hohhot	40.80	111.64
**6**	Jinan	36.66	117.05
**7**	Liulihe	39.58	116.00
**8**	Qinhuangdao	39.91	119.56
**9**	Shijiazhuang	38.03	114.54
**10**	Taiyuan	37.82	112.57
**11**	Tangshang	39.90	118.60
**12**	Tianjin	39.10	117.17
**13**	Xianghe	39.78	116.96
**14**	Yizhuang	39.80	116.51
**15**	Yufa	39.52	116.31
**16**	Zhengzhou	34.28	113.68

Table S2. List of the 34 monitoring sites in the United States.

Monitoring Sites in the United States			
No.	Site name	Latitude	Longitude
1	Parsons	39.09	-79.66
2	Prince Edward	37.17	-78.31
3	Perkinstown	45.21	-90.60
4	Rocky Mtn NP Collocated	40.28	-105.55
5	Sand Mountain	34.29	-85.97
6	Stockton	42.29	-90.00
7	Sequoia NP - Ash Mountain	36.49	-118.83
8	Wash. Crossing	40.31	-74.87
9	Yosemite NP - Turtleback Dome	37.71	-119.71
10	Abington	41.84	-72.01
11	Alhambra	38.87	-89.62
12	Arendtsville	39.92	-77.31
13	Beaufort	34.88	-76.62
14	Caddo Valley	34.18	-93.10
15	Cadiz	36.78	-87.85
16	Chiricahua NM	32.01	-109.39
17	Cherokee Nation	35.75	-94.67
18	Candor	35.26	-79.84
19	Coweeta	35.06	-83.43
20	Connecticut Hill	42.40	-76.65
21	Deer Creek	39.64	-83.26
22	Georgia Station	33.18	-84.41
23	Palo Duro	34.88	-101.67
24	Joshua Tree NP	34.07	-116.39
25	Bondville	40.05	-88.37
26	Great Smoky NP - Look Rock	35.63	-83.94
27	Indian River Lagoon	27.85	-80.46
28	Santee Sioux	42.83	-97.85
29	Beltsville	39.03	-76.82
30	Everglades NP	25.39	-80.68
31	Mount Rainier NP	46.76	-122.12
32	Kane Exp. Forest	41.60	-78.77
33	Konza Prairie	39.10	-96.61
34	Mackville	37.70	-85.05

Table S3. Distribution of observational cases in China in each month (with outliers removed)

Month	Jan.	Feb.	Mar.	Apr.	May	Jun.	Jul.	Aug.	Sep.	Oct.	Nov.	Dec.
Number of cases	104	144	176	157	268	184	182	111	30	45	134	231

Table S4. Summary of the inputs of Multivariable Taylor Series Method (MTSM) calculation. The unit of concentrations is μg m^{-3}, the RH is a relative number with no unit, and the unite of temperature is K. The values in "observation" group are the average values based on observation data, the values in "simulation group" are the average values based on CMAQ simulation data and the "Simulation, population-weighted" group is the population-weighted values based on CMAQ simulation data.

	**Observation**									
	Na^+	SO_4	TNH_3	TNO_3	TCl	Ca^{2+}	K^+	Mg^{2+}	RH	Temp
**US**	0.16	2.16	1.87	1.75	0.39	0.03	0.07	0.05	0.71	284.75
**China**	0.69	9.19	26.53	13.11	4.1	0.03	0.72	0.15	0.45	287.39
	**Simulation**									
	Na^+	SO_4	TNH_3	TNO_3	TCl	Ca^{2+}	K^+	Mg^{2+}	RH	Temp
**US**	0.03	0.85	0.56	0.75	0.11	0.02	0.02	0.01	0.72	285.96
**China**	0.08	1.95	3.05	2.82	0.11	0.05	0.11	0.02	0.72	280.98
	**Simulation, population-weighted**									
	Na^+	SO_4	TNH_3	TNO_3	TCl	Ca^{2+}	K^+	Mg^{2+}	RH	Temp
**US**	0.03	1.42	1.79	2.41	0.13	0.03	0.08	0.01	0.66	287.86
**China**	0.18	3.96	8.41	7.21	0.23	0.09	0.29	0.04	0.7	289.26
	**Sensitivity test, increasing concentrations with a constant factor and then changing the composition**									
	Na^+	SO_4	TNH_3	TNO_3	TCl	Ca^{2+}	K^+	Mg^{2+}	RH	Temp
**US, original**	0.16	2.16	1.87	1.75	0.39	0.03	0.07	0.05	0.71	284.75
**US composition, Chinese level**	1.33	18.19	15.74	17.42	3.28	0.22	0.62	0.43	0.71	284.75
**China**	0.69	9.19	26.53	13.11	4.1	0.03	0.72	0.15	0.71	284.75

Reference

Behera, S. N., Betha, R., and Balasubramanian, R.: Insights into Chemical Coupling among Acidic Gases, Ammonia and Secondary Inorganic Aerosols, Aerosol and Air Quality Research, 13, 1282-1296, 10.4209/aaqr.2012.11.0328, 2013.

Ding, J., Zhao, P., Su, J., Dong, Q., Du, X., and Zhang, Y.: Aerosol pH and its driving factors in Beijing, Atmos. Chem. Phys., 19, 7939-7954, 10.5194/acp-19-7939-2019, 2019.

Ge, Z., Wexler, A. S., and Johnston, M. V.: Deliquescence Behavior of Multicomponent Aerosols, The Journal of Physical Chemistry A, 102, 173-180, 10.1021/jp972396f, 1998.

Guo, H., Liu, J., Froyd, K. D., Roberts, J. M., Veres, P. R., Hayes, P. L., Jimenez, J. L., Nenes, A., and Weber, R. J.: Fine particle pH and gas–particle phase partitioning of inorganic species in Pasadena, California, during the 2010 CalNex campaign, Atmos. Chem. Phys., 17, 5703-5719, 10.5194/acp-17-5703-2017, 2017a.

Guo, H., Weber, R. J., and Nenes, A.: High levels of ammonia do not raise fine particle pH sufficiently to yield nitrogen oxide-dominated sulfate production, Scientific Reports, 7, 12109, 10.1038/s41598-017-11704-0, 2017b.

Tian, S., Pan, Y., and Wang, Y.: Ion balance and acidity of size-segregated particles during haze episodes in urban Beijing, Atmospheric Research, 201, 159-167, https://doi.org/10.1016/j.atmosres.2017.10.016, 2018.

Wang, Y., Chen, Y., Wu, Z., Shang, D., Bian, Y., Du, Z., Schmitt, S. H., Su, R., Gkatzelis, G. I., Schlag, P., Hohaus, T., Voliotis, A., Lu, K., Zeng, L., Zhao, C., Alfarra, M. R., McFiggans, G., Wiedensohler, A., Kiendler-Scharr, A., Zhang, Y., and Hu, M.: Mutual promotion between aerosol particle liquid water and particulate nitrate enhancement leads to severe nitrate-dominated particulate matter pollution and low visibility, Atmos. Chem. Phys., 20, 2161-2175, 10.5194/acp-20-2161-2020, 2020.

Xie, Y., Wang, G., Wang, X., Chen, J., Chen, Y., Tang, G., Wang, L., Ge, S., Xue, G., Wang, Y., and Gao, J.: Nitrate-dominated PM2.5 and elevation of particle pH observed in urban Beijing during the winter of 2017, Atmos. Chem. Phys., 20, 5019-5033, 10.5194/acp-20-5019-2020, 2020..

Zhao, M., Wang, S., Tan, J., Hua, Y., Wu, D., and Hao, J.: Variation of Urban Atmospheric Ammonia Pollution and its Relation with PM2.5 Chemical Property in Winter of Beijing, China, Aerosol and Air Quality Research, 16, 1390-1402, 10.4209/aaqr.2015.12.0699, 2016.

Zheng, G., Su, H., Wang, S., Andreae, M. O., Pöschl, U., and Cheng, Y.: Multiphase buffer theory explains contrasts in atmospheric aerosol acidity, Science, 369, 1374, 10.1126/science.aba3719, 2020.
